# Transcriptional adaptation of olfactory sensory neurons to GPCR identity and activity

**DOI:** 10.1038/s41467-022-30511-4

**Published:** 2022-05-25

**Authors:** Luis Flores Horgue, Alexis Assens, Leon Fodoulian, Leonardo Marconi, Joël Tuberosa, Alexander Haider, Madlaina Boillat, Alan Carleton, Ivan Rodriguez

**Affiliations:** 1 Department of Genetics and Evolution, Faculty of Sciences, University of Geneva, Geneva, Switzerland; 2 Department of Basic Neurosciences, Faculty of Medicine, University of Geneva, Geneva, Switzerland

**Keywords:** Genetics of the nervous system, Sensory processing, Cellular neuroscience

## Abstract

In mammals, chemoperception relies on a diverse set of neuronal sensors able to detect chemicals present in the environment, and to adapt to various levels of stimulation. The contribution of endogenous and external factors to these neuronal identities remains to be determined. Taking advantage of the parallel coding lines present in the olfactory system, we explored the potential variations of neuronal identities before and after olfactory experience. We found that at rest, the transcriptomic profiles of mouse olfactory sensory neuron populations are already divergent, specific to the olfactory receptor they express, and are associated with the sequence of these latter. These divergent profiles further evolve in response to the environment, as odorant exposure leads to reprogramming via the modulation of transcription. These findings highlight a broad range of sensory neuron identities that are present at rest and that adapt to the experience of the individual, thus adding to the complexity and flexibility of sensory coding.

## Abstract (web summary)

Olfaction in mammals relies on a toolbox composed of a varied set of sensory neurons. Here, the authors report in mice that this olfactory neuronal diversity relies both on the chemoreceptor that each neuron expresses and on the experience of this neuron.

## Introduction

Mammals use various sensory tools to build a representation of the outside world. A precise and robust representation being critical for survival and reproduction, we evolved a considerable number of different receptors able to respond to external stimuli. This is particularly true for chemical recognition, for which most of us benefit from large odorant chemoreceptor gene repertoires, that range in the hundreds in humans and dogs, up to over a thousand in mice and elephants^1–3^. Our understanding of olfactory information coding is based on the expression of a single chemoreceptor gene, stochastically chosen from a single allele, in each olfactory sensory neuron (OSN); this is referred to as singular expression^4–7^. Given the millions of OSNs present in a nasal cavity, large neuronal populations with identical agonist receptivity coexist with other populations that exhibit different response profiles. Olfactory receptors being able to bind various molecules and a volatile to be recognized by different receptors, to any given olfactory stimulus corresponds a specific pattern of activation. The term combinatorial coding has been coined to describe this encoding of chemical identities^8,9^. The odorant-dependent activation map is not just a concept, but is directly observable in the olfactory bulb, where axonal projections of OSNs coalesce into neuropil-rich structures, the glomeruli, that are each innervated by a specific sensory population. Following this first relay of olfactory processing and after being transmitted to cortical areas, olfactory information often translates into a percept, a smell. A series of parallel and invariable coding lines, defined by the expressed odorant receptor (OR) (and a few guidance molecules that may be differentially expressed between populations), is thus at the heart of the way we understand olfaction.

The ability of neurons to adapt, to respond dynamically to the environment, both during development and later, is critical^10^. In the central nervous system, adaptive and compensatory mechanisms that control neural activity, for example through the modulation of synaptic efficacy and membrane excitability, allow homeostasis maintenance within the system upon perturbations^11^. Various mechanisms answering this need have been selected during evolution, among which the repression or activation of specific genes^10,12,13^. In a peripheral sensory tool such as the olfactory system, which is in direct contact with highly variable environments, rapid and long-term sensory adaptation and compensatory mechanisms may be of particular importance. Activity-regulated transcription in the olfactory system has been addressed in the past by various groups in the context of axon guidance mechanisms, OSN survival and adaptation to the environment^14–24^. Almost without exception, these approaches have involved the silencing of neuronal activity via naris occlusion or activity mutants, thus adding various confounding factors to odorant-induced activity. Due to a lack of technology available at the time, most of these approaches explored the system at the level of the whole olfactory mucosa, precluding an evaluation of the response to a specific ligand of specific neurons or specific neuronal populations expressing a given OR. We recently reported that following in vivo odorant exposure of OSNs in the mouse, a decrease in the amount of mRNA encoding for the OR gene expressed by the activated neurons takes place^25^. Whether this odorant-induced decrease of mRNA concentration is limited to the receptor mRNA, or whether it is part of a broader activity-induced transcriptional alteration that could modulate the neuron’s genetic identity, remains to be determined.

In this work, we explored the transcriptional identities of olfactory neuronal populations and their modulation upon activation. We characterized the transcriptomes of several thousand mouse OSNs and uncovered a variability of profiles, each defined by the differential expression of numerous genes, and dependent on the expressed chemoreceptor. Following neuronal activation, we found that a second layer of transcriptome variability is added to this initial landscape. It results from the transcriptional modulation of hundreds of genes and represents a potential tool to adapt sensory responses to upcoming signals.

## Results

### Variable transcriptomes among OSN populations at rest

The main determinant of an OSN identity is the OR gene it expresses. In addition to this functional characteristic, a few genes have been described to be unequally expressed in different olfactory populations. These latter are however thought to be shared by large numbers of OSN populations (that is populations expressing different ORs), and to be involved in the topographic organization of the olfactory sensory mucosa, in which neurons expressing a given OR are restricted to specific zones.

**Fig. 1:**
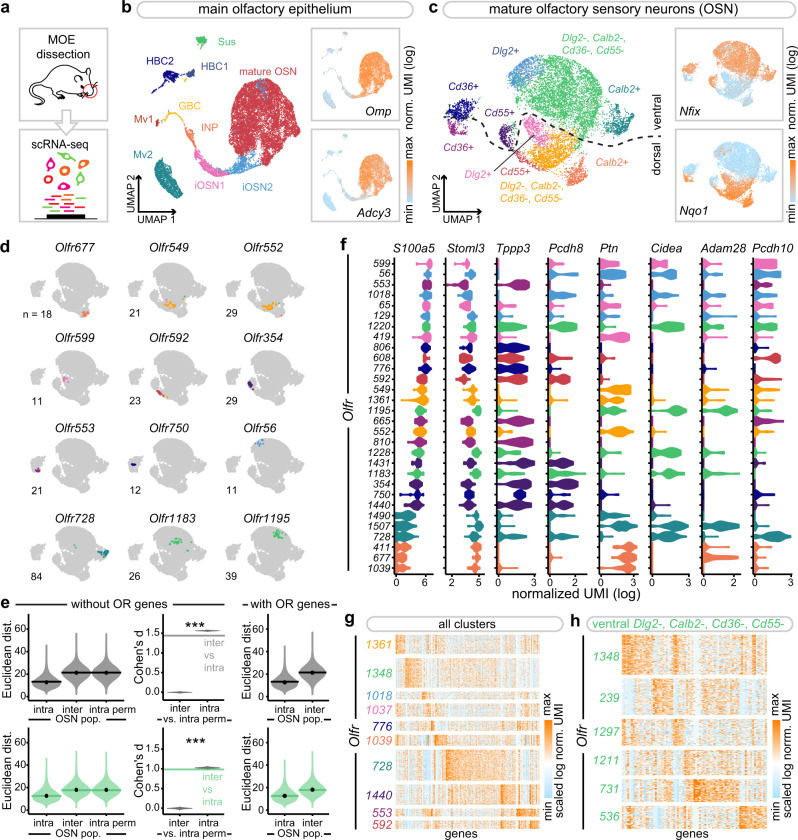
Variable odorant receptor-associated transcriptomes among olfactory sensory populations. **a** Schematic of the approach. **b** Main olfactory epithelium (MOE) cell clusters on a UMAP plot. *Inset*: normalized expression levels of two mature olfactory sensory neuron (OSN) gene markers, *Omp* and *Adcy3*. HBC horizontal basal cells, GBC globose basal cells, Sus sustentacular cells, Mv microvillar cells, INP immediate neuronal precursors, iOSN immature OSNs. **c** Mature OSN cell clusters on a UMAP plot computed after removing the olfactory receptor gene counts from the data. Inset: normalized expression levels of *Nfix* and *Nqo1* (markers of neurons located ventrally and dorsally). **d** Dispersion of OSN populations on the UMAP plot shown in (**c**). Colors indicate the cluster to which the cells pertain. **e** (left) Density distribution of transcriptomic Euclidean distances (computed on the first 15 PCs) between pairs of OSNs expressing the same receptor (intra, *n* = 97,999), different receptors (inter, *n* = 45,393,492), and the same receptor after permutation of all receptor identities prior to distance calculation (intra perm, *n* = 97,999,000, see methods). Horizontal bars correspond to mean values and dots correspond to median values. (middle) Range of Cohen’s d values calculated between the distribution of intra or inter OSN population pairwise transcriptomic Euclidean distances and each of the distributions of distances after permutation of receptor identities (*n* = 1000 per violin plot). The horizontal bar corresponds to the Cohen’s d value computed between the distributions of intra and inter OSN population pairwise transcriptomic Euclidean distances. (right) Density distribution of transcriptomic Euclidean distances (computed on the first 14 PCs) between pairs of OSNs expressing the same receptor (intra) or different receptors (inter). Data are plotted for all OSNs (top) or ventral cluster OSNs (bottom). ***p < 0.001; two-sample Kolmogorov–Smirnov test. **f** Mature OSN population-specific distribution of selected markers (log-normalized UMI). OSN populations are ordered by their mean expression of *S100a5*. Colors indicate the cluster to which the majority of the cells from the given population pertain. Exact *p* values are provided in Supplementary Table 1. **g**, **h** Expression levels of specific gene markers of the largest mature OSN populations selected from each cluster (**g**) or from the ventral *Dlg2*-, *Calb2*-, *Cd36*- and *Cd55*- clusters (**h**). Source data are provided as a Source Data file.

As an initial approach to determine potential differences in the identity of the various sensory neurons populating the main olfactory epithelium, we performed a single-cell RNA-seq of dissociated cells extracted from the nasal cavity of 8-week-old male mice (Fig. 1a). The data were clustered and visualized on a UMAP plot (Fig. 1b and Supplementary Fig. 1). We obtained a total of 15,859 cells, from which mature OSNs (expressing the *Omp* and *Adcy3* genes) were readily distinguishable from immature neurons and non-neuronal cells (Fig. 1b and Supplementary Fig. 2a), in agreement with previous observations^26^. A total of 9,539 mature OSNs (composed of 798 olfactory sensory populations of at least three neurons (Supplementary Fig. 2d)) expressing the olfactory marker gene *Omp* were then selected, and clustered again. Specific subclusters were observed (Fig. 1c and Supplementary Fig. 2b), that were defined by the expression of marker genes (*Calb2*, *Cd36* and others), and that showed a clear separation between sensory neurons located ventrally (*Nfix*+) and dorsally (*Nqo1*+) in the nasal cavity (OSNs expressing specific ORs are unequally scattered across the nasal epithelium^27^). We then explored the potential transcriptional proximity of neurons expressing the same OR. Their positions were visualized on the UMAP plot, which revealed a striking grouping of each of the different neuronal populations (Fig. 1d and Supplementary Fig. 4), ranging from dense transcriptomic clustering of some populations (such as those expressing *Olfr354* or *Olf553*), to populations exhibiting a larger variance in gene expression (such as *Olfr1183*). To quantify this observation, we measured the pairwise transcriptomic distances (computed on the PC space used for clustering and UMAP plot generation) between pairs of sensory neurons expressing the same receptor (intra) and found that neurons expressing the same receptor were significantly more similar transcriptionally than those expressing different receptors (inter) (Fig. 1e, Supplementary Figs. 6 and  7). To determine the potential role played by the expression of the ORs themselves in this clustering, this latter was performed without taking the OR gene expression data into account. Remarkably, the grouping of populations expressing the same chemoreceptor, irrespective of whether the analysis was performed on the whole sensory population or a given subcluster (Fig. 1e and Supplementary Fig. 5), was maintained. These results were stable over a wide range of number of top PCs chosen for downstream analyses (Supplementary Fig. 3). To explore this OR-associated population specificity, we identified specific genes that were differentially expressed between the different neuronal populations (Fig. 1f and Supplementary Fig. 2c). These included genes that were either transcribed or whose transcripts were absent in the different populations such as *Cidea*, or that were expressed in a graded manner across most populations, such as *S100a5*. These transcriptomic profiles were not merely reflecting different general types of sensory neurons, since they were not only observed to be different between subclusters, but also within subclusters (Fig. 1g, h). At rest, that is without active olfactory stimulation, a transcriptomic code thus characterizes each OR-defined population.

### Transcriptomic proximity versus olfactory receptor identity

What may determine the transcriptional distance between two neuronal populations expressing different ORs? Considering OSN maturation as a series of differentiation steps that at each increment determine more and more specific identities^26,28^, transcriptomic distances between two mature sensory neuron populations may reflect how many of these steps they shared. If transcriptomic identity is largely determined prior to odorant gene choice, the closeness between two populations should be mirrored by their use of the same OR gene cis-regulatory element. These elements are known to be necessary for the choice of specific sets of OR genes and control small clusters of adjacent OR genes^29–32^. Alternatively, or in addition to this first hypothesis, one could envisage a direct role played by the receptor itself, which may define a basal activity level in neurons for example, and a corresponding transcriptomic profile.

**Fig. 2:**
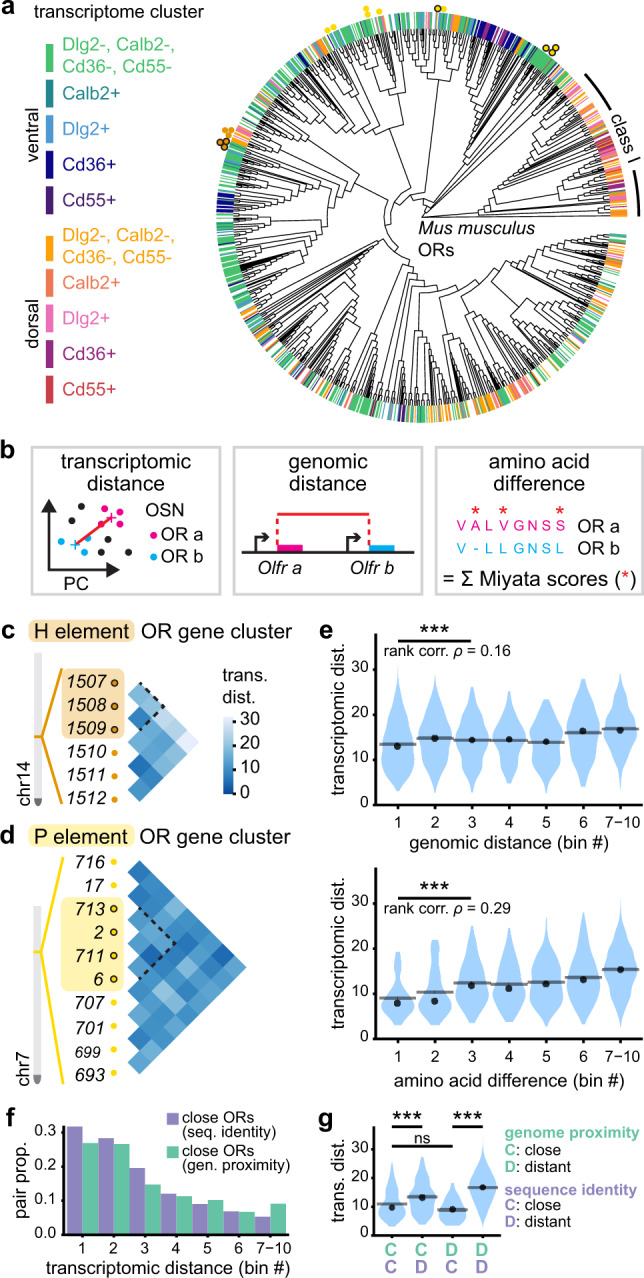
Association between transcriptomic distances and odorant receptor similarity levels. **a** 1152 mouse odorant receptor (OR) phylogeny. Colored bars show the transcriptomic identity of the olfactory sensory neuron (OSN) population expressing a given OR. Colored dots indicate that the corresponding OR belongs to the gene cluster analyzed in (**c**, **d**). **b** Schematics of the different metrics used to express distance between pairs of OSN populations. From left to right, transcriptomic distance (Euclidean distance between centroids of OSN population transcriptomes in the PCA space), genomic distance (base pairs between OR gene start codons), amino acid difference (sum of Miyata amino acid replacement scores between OR protein sequences). **c** Pairwise transcriptomic distances between OSN populations expressing OR genes in the same cluster on chromosome 14. The orange box highlights H element-regulated OR genes. The dotted line in the pairwise distance matrix encompasses comparisons between OR genes under control of the H element. **d** Same analysis as in (**c**) but for OR genes under the control of the P element, on chromosome 7. **e** Distribution of transcriptomic distances per bins of either genomic distances (top) or amino acid differences (bottom), for all pairs of ORs belonging to the same class and the same gene cluster (*n* = 3602 pairs). Transcriptomic distances and genomic distances: Spearman’s rank correlation *ρ* = 0.16, ***p < 0.001, *n* = 2598 pairs. Transcriptomic distances and amino acid differences: Spearman’s rank correlation *ρ* = 0.29, ***p < 0.001, *n* = 222 pairs. Correlation scores (*ρ*) and associated *p* values between the transcriptomic distances and each of the different metrics was calculated for the pairs included in the three first bins. **f** Proportions of closely related pairs of OSN populations across bins of transcriptomic distances. Close pairs are defined depending on either their genomic distance or their amino acid differences. **g** Transcriptomic distance distribution of four categories of OSN population pairs defined by a combination of their genomic distances and amino acid differences. ***p < 0.001, ns *p* > 0.05; two-sided Wilcoxon rank test, *p* values were adjusted with a Bonferroni correction. Exact *p* values are provided in Supplementary Table 1. Source data are provided as a Source Data file.

We tested the first hypothesis by evaluating whether OR genes sharing a common enhancer are transcriptionally closer to each other than to those under the control of other cis-regulatory elements. We took advantage of two well defined cis-regulatory elements acting on mouse OR genes (the H and P elements), whose OR gene targets are quite dissimilar (Fig. 2a–d), and have been well described^29–32^. Calculating a centroid-based Euclidean distance between OSN population transcriptomes (Fig. 2b and Supplementary Fig. 8a), we evaluated the similarity between the transcriptomes of sensory neuron subpopulations expressing OR genes under the control of the same cis-regulatory elements, as well as their neighbors (Fig. 2c, d). No increase in transcriptomic similarity was observed between genes under the control of the H or P elements. To further explore this hypothesis, we took a global approach based on the possible link between the genomic distance separating OR genes and their difference in transcriptomic identity, the idea being again that since olfactory cis-regulatory elements act on adjacent genes, those located in proximity may also result closer transcriptionally. Pairwise genomic distances between OR genes (Fig. 2b) were calculated for all pairs of genes located in the same gene cluster and compared to the transcriptomic distance between the OSN populations expressing these ORs. A weak positive association between genomic and transcriptomic proximity was observed in the first 30% of the genomic distance value range (Fig. 2e and Supplementary Fig. 8f). This association decreased when using a binning based on the intergenic distance between OR genes (Supplementary Fig. 8c, d) and including approximately the first 10% of the genomic distance value range. This relationship was more visible when considering different equal width bins of the observed range of transcriptomic distances, in relation to the proportion of pairs of neighboring genes (Fig. 2f and Supplementary Fig. 8b, c).

We then evaluated our second hypothesis, which proposes that the ORs themselves define transcriptomic profiles. We determined the levels of sequence homology between ORs using Miyata scores (Fig. 2b and Supplementary Fig. 8e) and tested their potential association with the transcriptomic distances between the corresponding neuronal populations. We found a positive association between transcriptomic proximity and OR sequence similarity in the first 30% of the amino acid difference value range (Fig. 2e and Supplementary Fig. 8g), that was further supported by a clear overrepresentation of similar OR pairs in sensory populations that are transcriptionally close (Fig. 2f and Supplementary Fig. 8e). Given this last observation and knowing that OR genes tend to duplicate in cis, it is likely that sequence identity is a confounding factor when measuring genomic proximity. To reevaluate whether genomic proximity plays indeed a role in addition to receptor identity, we took advantage of evolutionary accidents that led duplicated OR genes to land in close vicinity or distantly from their princeps allele. We thus evaluated the potential differences in transcriptional proximity between neuronal populations expressing ORs that are similar and associated in the genome, and neuronal populations expressing ORs that are similar but located in different gene clusters. In parallel, we evaluated the transcriptomic distances of neuronal populations expressing ORs that are dissimilar and are associated in the genome (Fig. 2g). We found no significant difference in the average transcriptional distance between neurons expressing similar OR genes, whether these genes are located in proximity or are distant from each other. On the contrary, we found an increase in transcriptomic distances between dissimilar and similar ORs associated in the genome, supporting our second hypothesis, that is a role played by the OR identity in transcriptome determination.

### Activity-induced transcriptomic modulation in defined OSN populations

**Fig. 3:**
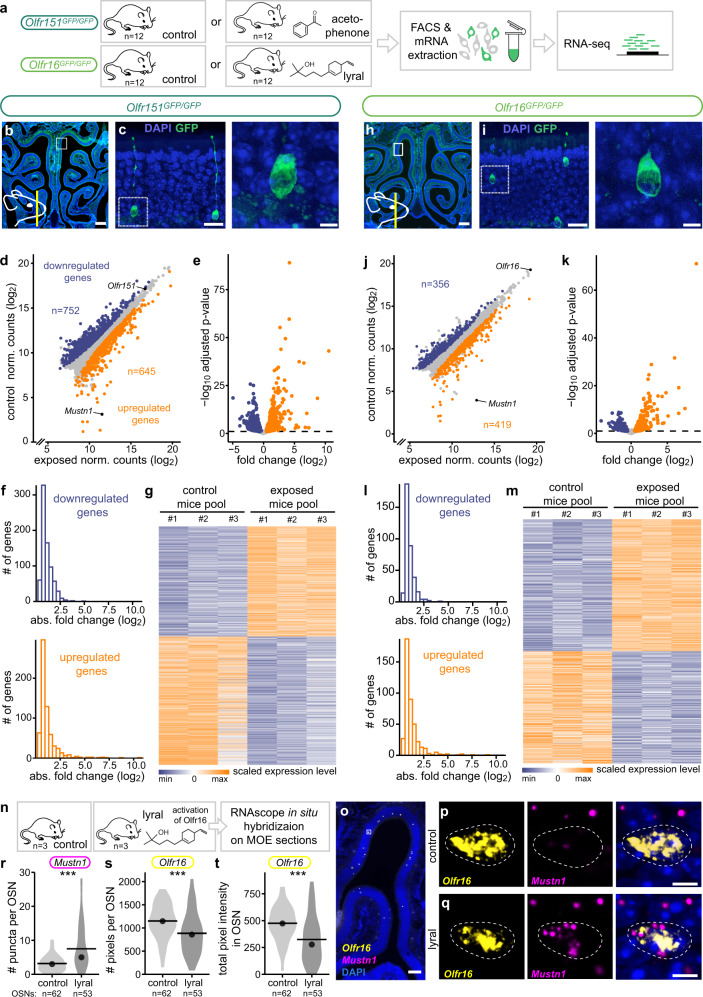
Odorant-induced transcriptomic modulations. **a** Schematic of the experiment. After being exposed to their cognate ligand for 5 h, fluorescent neurons from *Olfr151*^*GFP/GFP*^ and *Olfr16*^*GFP/GFP*^ mice were FAC-sorted and total mRNA was sequenced. **b**, **c** Coronal section of an *Olfr151*^GFP/GFP^ mouse main olfactory epithelium (MOE) containing GFP-expressing neurons (green, endogenous GFP) and stained with DAPI (blue). The schematic in (**b**) indicates the antero-posterior position of the section and the white square the region magnified in (**c**). Scale bars, 0.5 mm (**b**), 20 μm (**c**, left) and 5 μm (**c**, right). **d** Scatter plot showing mean normalized counts resulting from the differential expression analysis (exposed versus non-exposed *Olfr151*^*GFP/GFP*^ mice). Blue and orange dots: significantly downregulated and upregulated genes, respectively. Black dots highlight *Mustn1* and *Olfr151*. **e** Volcano plot showing differentially expressed genes (DEGs) between exposed and non-exposed *Olfr151*^*GFP/GFP*^ mice. The x-axis is the log_2_ scale of the gene expression fold change. Negative values indicate downregulation and positive values upregulation. The y-axis is the minus log_10_ scale of the adjusted *p* values. **f** Distribution of the DEGs between exposed and non-exposed *Olfr151*^*GFP/GFP*^ mice based on their fold change (log_2_). **g** Heatmap showing DEGs between exposed and non-exposed *Olfr151*^*GFP/GFP*^ mice. **h**–**m** Same analyses as in (**b**–**g**) but with *Olfr16*^GFP/GFP^ exposed and non-exposed mice. **n** Schematic of the experiment. After being exposed to lyral for 5 h, coronal sections of the MOE were processed for RNAscope in situ hybridization (also see Supplementary Fig. 10). **o** Coronal section of the MOE labeled with probes against *Olfr16* (yellow) and *Mustn1* (magenta) and counterstained with DAPI (blue). White square highlights a region of interest with an *Olfr16*-transcribing cell. Scale bar, 100 µm. **p**, **q** Magnification of a cell expressing *Olfr16* in a control mouse (**p**) and in a mouse exposed to the Olfr16 agonist lyral (**q**). Channels are shown separately and together with the DAPI staining. Scale bar, 5 µm. **r**–**t** Quantification of *Mustn1* (**r**) and *Olfr16* (**s**, **t**) transcription. Horizontal bars correspond to mean values and dots correspond to median values. ***p < 0.001; two-sided Wilcoxon signed rank test. Exact *p* values are provided in Supplementary Table 1. Source data are provided as a Source Data file.

Following the identification of specific transcriptomic identities characterizing the different olfactory neuronal populations at rest, we explored their potential evolution after agonist activation. To address this question, we determined the transcriptome of defined and well described neuronal populations following exposure to odorants (Fig. 3a). We used two knockin mouse lines, *Olfr151*^*GFP/GFP*^ and *Olfr16*^*GFP/GFP*^, in which OSNs expressing the *Olfr151* (*M71*) and the *Olfr16* (*MOR23*) OR genes are modified such that when transcribed, a green fluorophore is coexpressed. These olfactory receptors have different sequences (Miyata Score=273.24), are expressed in different subzones of the olfactory epithelium (Fig. 3b, h), are expressed in different basoapical layers (Fig. 3c, i), and respond to different agonists. 12 live and freely moving mice were exposed for 5 h to acetophenone and lyral, two known agonists of Olfr151 and Olfr16, respectively^33,34^ (Fig. 3a). To extract the most possible transcriptomic information, we did not opt here for a scRNA-seq approach but rather for the bulk sequencing of purified neuronal populations. Following exposure, fluorescent *Olfr151*- and *Olfr16*-expressing neurons were isolated by FACS, and their transcriptomes were determined and analyzed. In all conditions, high amounts of transcripts of the OR gene corresponding to the targeted population were detected and sequence reads from other OR genes were almost absent (Supplementary Fig. 9). Significant and robust transcriptomic modulations were observed for both olfactory populations after agonist exposure, with 645 and 752 genes upregulated and downregulated respectively for *Olfr151*-transcribing neurons, and 419 genes upregulated and 356 downregulated for *Olfr16*-transcribing neurons (Fig. 3d–g, j–m and Supplementary Data 1). The fold-change modulation ranged from 0.032 to 1505 and from 0.056 to 517 in the *Olfr151*- and *Olfr16*-transcribing populations, respectively (Fig. 3f, l).

To confirm the specificity of the transcriptional modulation, we performed single-molecule RNA in situ hybridization (via RNAscope) to evaluate expression levels of two genes of interest: *Mustn1*, which we found to be highly upregulated in the FACS-seq experiments, and *Olfr16*, which we expect to be downregulated upon activation (see Fig. 3d, e, j, k). In mice exposed to lyral, the *Olfr16-*neuronal population showed significant upregulation of *Mustn1*, while the transcripts of the OR gene were downregulated (Fig. 3n–t). Conversely, when mice were exposed to acetophenone, which is not a ligand for Olfr16, there was no transcriptional modulation occurring in the *Olfr16*-transcribing population (Supplementary Fig. 10). This confirms that the transcriptional modulations observed in the FACS-seq analysis are indeed triggered by the binding of agonists to the receptor defining the population, and not by aspecific activity within the olfactory sensory epithelium.

### Common activity-induced transcriptomic adaptation in different OSN populations

**Fig. 4:**
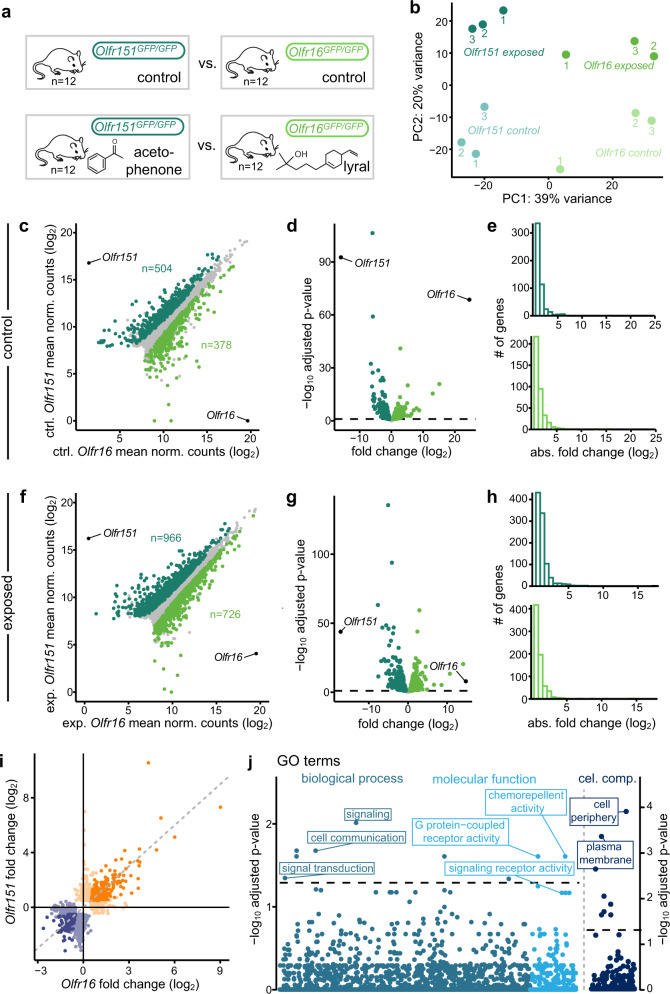
Shared odorant-induced transcriptomic modulations between different olfactory populations. **a** Schematic of the experiment. After being exposed to their cognate ligand, fluorescent neurons from *Olfr151*^*GFP/GFP*^ and *Olfr16*^*GFP/GFP*^ mice were FAC-sorted and total mRNA was sequenced. *n* = 3 × 4 mice/condition. **b** Two dimensional PCA representing the differences in gene expression between two populations of olfactory sensory neurons (OSNs) in their basal state (non-exposed) and after exposure to their cognate ligand. Each dot represents a pool of 4 mice. **c** Scatter plot showing the differential expression analysis between *Olfr15-* and *Olfr16-*transcribing neurons in their basal state. Dark and light green dots: genes expressed significantly higher in *Olfr15*- and in *Olfr16*-transcription neurons, respectively. Black dots highlight *Olfr151* and *Olfr16*. **d** Volcano plot showing differentially expressed genes in *Olfr151*- and in *Olfr16*-transcribing neurons in their basal state. Dark and light green dots: genes expressed significantly higher in *Olfr15*- and in *Olfr16*-transcribing neurons, respectively. Black dots highlight *Olfr151* and *Olfr16*. **e** Distribution of the differentially expressed genes between *Olfr151*- and in *Olfr16*-transcribing neurons in their basal state. **f** Scatter plot showing the differential expression analysis between *Olfr151-* and *Olfr16-*transcribing neurons after exposure to their respective cognate ligands. Dark and light green dots: genes expressed significantly higher in *Olfr151*- and in *Olfr16*-transcribing neurons after activation, respectively. **g** Volcano plot showing differentially expressed genes in *Olfr151*- and in *Olfr16*-transcribing neurons after agonist exposure. Dark and light green dots: genes expressed significantly higher in *Olfr15*- and in *Olfr16*-transcribing neurons, respectively. **h** Distribution of the differentially expressed genes between *Olfr151*- and in *Olfr16*-transcribing neurons after agonist exposure. **i** Volcano plot showing differentially expressed genes in *Olfr151*- and in *Olfr16*-transcribing neurons after exposure to their respective cognate ligands. Orange and blue dots: genes significantly upregulated and downregulated in both *Olfr151*- and *Olfr16*-transcribing neurons, respectively, after agonist exposure. Light orange and blue dots represent genes modulated in either *Olfr151*- and in *Olfr16*-transcribing neurons, respectively. **j** Gene Ontology analysis of the common differentially expressed genes in *Olfr151*- and in *Olfr16*-transcribing neurons. The dashed line corresponds to the significant threshold (adjusted *p* values < 0.05). Raw *p* values were extracted with Fisher’s exact test and then adjusted for multiple comparisons with the Benjamini–Hochberg correction. Source data are provided as a Source Data file.

Taking advantage of our double approach and to potentially extract general rules, we compared the transcriptomic modulations of the *Olfr151-* and *Olfr16*-transcribing populations after agonist exposure (Fig. 4a). In accordance with our initial observations describing significant transcriptomic distances between populations expressing different receptors, we first observed that the transcriptomes of *Olfr151*- and *Olfr16*-transcribing neurons were significantly dissimilar (882 genes were differentially expressed between the two neuron populations (Fig. 4b–e). Among the differentially expressed genes (DEGs) between O*lfr151-* and *Olfr16-*transcribing populations, several genes related to axon guidance were identified, such as *Pcdh7*, *Pcdh9, Nrp1*, and *Sema7A* (Supplementary Data 2).

We then compared the activity-induced modulated genes between the *Olfr151*- and *Olfr16-*transcribing populations (Fig. 4f–h), that without surprise, showed again a significant difference between populations. A principal component analysis (PCA) of the Olfr151 and Olfr16 transcriptomic sets, before and after agonist exposure, showed 39% of variance explained by cell identity, and 20% of variance by activity. The overlap between the activity-induced responses of the *Olf151*- and *Olfr16*-transcribing populations was further explored by comparing their potentially common down and upregulated genes. A significant proportion of these genes were shared, 215 (51.3%) and 134 (37.6%) of them being commonly upregulated and downregulated, respectively (Fig. 4i).

In both *Olfr151*- and *Olfr16*-activated populations, the dispersion of downregulated genes decreased, while it increased for upregulated genes; this was also true for the genes shared by both populations (Supplementary Fig. 11). We then aimed to functionally interpret our data by attributing Gene Ontology (GO) terms to the activity-dependent modulated genes common to the *Olfr151-* and *Olfr16*-transcribing populations. Among the significantly enriched terms, we found “signaling” and “G-protein-coupled receptor activity” in the GO biological processes terms, “molecular transducer activity” in the GO molecular functions category, and “plasma membrane” and “cell periphery” in the GO cellular components category (Fig. 4j and Supplementary Table 2). A pattern thus emerged, pointing to actors of the transduction cascade being modulated by agonist exposure.

We finally looked at genes that were modulated after activation in one OSN population and not in the other (fold change after activation < 0.5 for downregulated genes and >2 for upregulated genes, Supplementary Data 3). Among these genes, we observed that a large proportion encoded transcription factors (8.2% and 7.1% for *Olfr151*- and *Olfr16-*transcribing populations, respectively, to be compared to 5% observed in commonly modulated genes).

### Modulation of transcription following odorant exposure

**Fig. 5:**
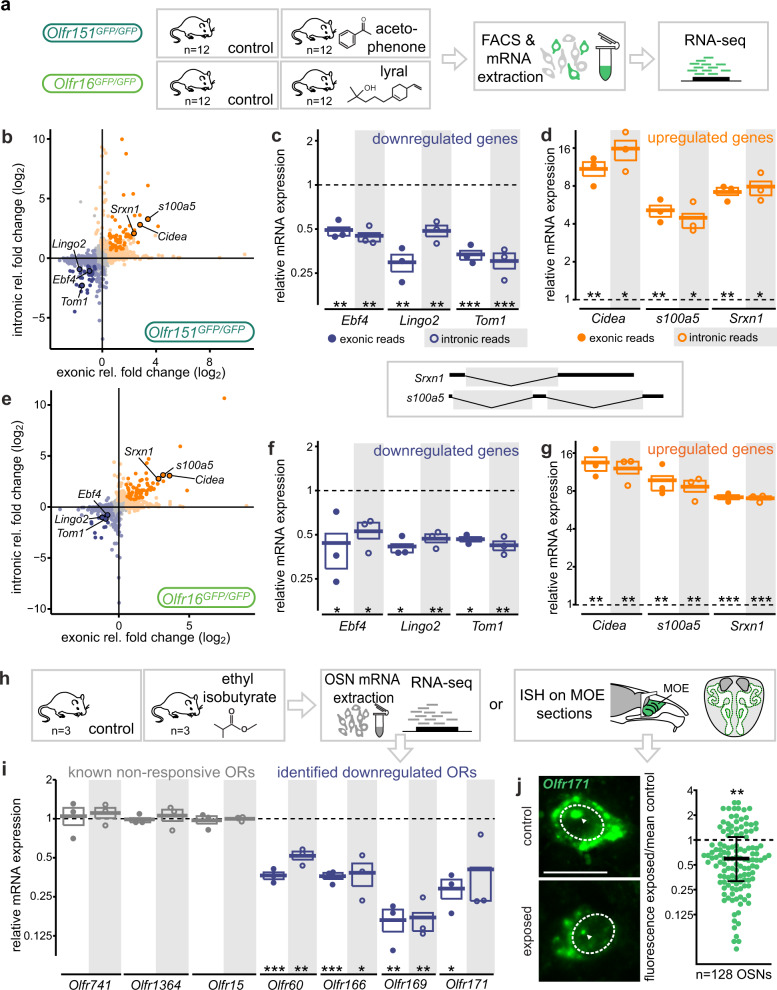
Odorant-induced modulations of mRNA levels result from transcriptional regulation. **a** Schematic of the experiment. After exposure to their cognate ligand, fluorescent neurons from *Olfr151*^*GFP/GFP*^ and *Olfr16*^*GFP/GFP*^ mice were FAC-sorted and total mRNA sequenced. *n* = 3 × 4 mice/condition. **b** Fold changes observed using exonic or intronic reads of FAC-sorted *Olfr151*-transcribing neurons. Each gene is represented by a dot. **c**, **d** Examples of downregulated (**c**) and upregulated (**d**) genes in *Olfr151*-transcribing neurons after acetophenone exposure (shown in (**b**)). Expression levels in exposed samples relative to control conditions measured using exonic and intronic sequence reads. The dotted line represents the relative expression of non-exposed control samples and each dot represents the relative expression of an exposed sample. Box limits represent Q1 to Q3, the line represents the mean. FDR adjusted *p* values: *p < 0.1; **p < 0.01; ***p < 0.001, two-sided two-sample independent *t* test. **e**–**g** Same as (**b**–**d**) but with FAC-sorted *Olfr16*-expressing neurons after lyral exposure. **h** Schematic describing exposure of wild type mice to ethyl isobutyrate, followed by transcriptomic (**i**) or in situ hybridization (**j**) analyses. **i** The downregulation of four odorant receptor transcripts (corresponding to receptors activated by ethyl isobutyrate) was evaluated at the level of both intronic and exonic reads. The dotted line represents the relative expression of control samples, each dot corresponds to one exposed mouse. Box limits represent Q1 to Q3, the line represents the mean. FDR adjusted *p* values: *p < 0.1; **p < 0.01; ***p < 0.001, two-sided two-sample independent *t* test. **j** Left, representative images of a control (top) and ethyl isobutyrate-exposed (bottom) olfactory sensory neurons, hybridized with an *Olfr171* probe. Dashed lines delimit the nucleus, and arrowheads point towards the site of transcription of *Olfr171*. Scale bar, 10 μm. Right: fluorescence intensity within the transcription foci. Each dot represents an ethyl isobutyrate-exposed neuron. Fluorescence intensity was divided by the mean of all control neurons, whose relative expression is represented by the dotted line. The black bar represents the median and the error bars Q1 to Q3. **p = 0.0017, unpaired two-sided *t* test with Welch’s correction. All the exact *p* values are provided in Supplementary Table 1. Source data are provided as a Source Data file.

Modulations in mRNA concentration may result from various processes. Among them and first in line, the regulation of transcriptional activity and the modulation of mRNA half-life. Our previous work has pointed to a rapid downregulation of OR gene mRNA concentration following odor exposure (as fast as 20 min), and in some cases an almost absence of OR mRNA 5 h after stimulation^25^. This nearly immediate modulation and complete loss of messenger is suggestive, or at least compatible with an active degradation of cytosolic mRNAs. To explore this question, we took advantage of the different characteristics of nascent and mature mRNAs, namely the presence and lack of intronic sequences, respectively. We first analyzed the exonic versus intronic reads of the modulated genes that we identified after odorant exposure of *Olfr151*- and *Olfr16*-transcribing neurons (Fig. 5a). Following agonist exposure, a large portion of the genes whose modulation was determined after analysis of exonic reads, were also modulated, both up and down and in both *Olfr151*- and *Olfr16*-transcribing neurons, when restricting the analysis to intronic reads (Fig. 5b–g and Supplementary Fig. 12). To further explore this question, we exposed wild type mice to ethyl isobutyrate (Fig. 5h), an agonist for which we previously determined a set of highly responsive OR genes, among which *Olfr60*, *Olfr166*, *Olfr169*, and *Olfr171*, whose corresponding mRNA concentrations drastically decrease after exposure^25^. We evaluated this potential modulation at the level of nascent mRNAs. We observed a downregulation of OR mRNAs that was similar using exonic and intronic reads as readouts (Fig. 5i) (although, for some OR genes, the number of intronic reads in the bulk RNA-seq was too low to evaluate differential expression (Supplementary Fig. 13)). Finally, we looked at the cellular localization of transcripts (Fig. 5h). We took advantage of the high level of OR transcription that makes nascent transcripts easily visualized using in situ hybridization. Since OR genes are transcribed monoallelically, a single nuclear transcriptional spot corresponding to their expressed OR gene is observed in the nucleus of each sensory neuron (Fig. 5j). We exposed wild type mice to ethyl isobutyrate and performed in situ hybridizations with a probe specific for *Olfr171*, whose mRNA we previously showed to be modulated following ethyl isobutyrate stimulation. We quantified the intensity of the nuclear signal, before and after agonist exposure, and found a significant decrease in signal intensity after ethyl isobutyrate exposure (Fig. 5j). Taken together, these data all point to an odorant-induced modulation of transcription, and not to a mechanism involving degradation or stabilization of mature mRNAs.

## Discussion

In this work, we took advantage of the unique opportunity provided by the mouse olfactory epithelium, which contains hundreds of singular cell subpopulations, each defined by the expression of a known chemoreceptor gene, and activable at will. We first characterized the transcriptomes of these subpopulations in a basal state, at rest, that is in mice living in their usual environment without exposure to specific olfactory stimuli. Second, by activating defined OSN subpopulations in vivo using odorants, we further explored how these neurons adapt to environmental changes. We found that the transcriptomic identity of mouse OSNs is remarkably variable, a variability that results from the apparent interplay of two dimensions: a first, steady state transcriptomic identity that characterizes each OSN population, to which a second is added, following the recent odor-driven activity of the sensory neuron.

What does determine the transcriptomic identities of OSN populations? It is the expressed OR that appears to be the main factor in the establishment of these identities. Our data indeed show that transcriptomic similarities are better predicted by receptor similarities than by shared OR regulatory sequences. In agreement with our data, the importance of the OR in the establishment of the transcriptional identity was shown in a recently published manuscript^35^.

We showed here that odorant recognition triggers OSN transcriptomic modulation, that not only includes the downregulation of the expressed OR genes^25^, but the up- and downregulation of hundreds of additional ones. By comparing the agonist response of two olfactory populations expressing different ORs and responding to different odorants, we found a significant overlap between activity-modulated genes. These represent late responsive genes (in opposition to early response genes), typically encoding proteins that regulate dendritic growth, synapse elimination or spine maturation^10^. For example, a gene that was significantly upregulated in both *Olfr151*- and *Olfr16*-populations upon activation was the late responsive gene *Mustn1*, whose transcription is regulated by immediate early genes including *c-Fos* and *JunD*^36^. *Mustn1* may constitute an activity marker in OSNs, although additional experiments would be required to generalize this observation. More generally, in our case most modulated genes pertain to signal transduction categories, which possibly reflects the peculiarly ordered and relatively invariant olfactory circuitry. Interestingly, among the main genes involved in the olfactory transduction cascade, including those coding for ORs, *GnaI*, *Adcy3*, *Cnga2*, *Cngb1b*, or *Cnga4*, none were upregulated after odorant-mediated activation, and most were downregulated. This parallels the data presented by Tsukahara et al. in which most of these genes were also found modulated after naris occlusion and long-term odorant exposure^35^. Such transcriptomic contraction affecting all members of the olfactory cascade suggests a functional transcriptional adaptation, leading in the case of a sustained odorant exposure to a decreased response to the experienced odor. Such an adaptive mechanism would not be surprising, since functional plasticity of sensory inputs mediated by olfactory stimulation has been observed^37^.

We also report different transcriptomic identities of OSN populations at rest (that is in the absence of actively exposing mice to odors). In this almost silent state, how does the olfactory receptor translate its identity to the OSNs that express it? One may argue, as Tsukahara et al.^35^ that some OSNs expressing specific ORs will fire in a nose even without experimental stimulation, as some volatile molecules are always present in the environment. But we rather suggest that there is an inherent activity state that may characterize each sensory population at rest, that translates into a specific transcriptomic identity. This last view is supported by the observation that the elimination of odorant-induced activity through naris occlusion does not abolish transcriptomic variability between OSN populations^35^. Moreover, also supporting this view, it was previously shown that OSN populations expressing different ORs exhibit different basal activity levels, that is levels of basal transduction activity and fluctuation of currents, which drive concomitant firing of action potentials^38,39^. Even in the absence of odor-stimulation, OSNs are indeed characterized by high adenylate cyclase 3 activity and by large fluctuations in cAMP levels^39,40^, both of which are suggestive of intrinsic OR activity. An interesting candidate mechanism for the generation of such odorant receptor-dependent and odorant-independent graded cellular states is the agonist-independent natural basal activity of G-coupled receptors (GPCRs). Both GPCRs and other ligand-activated receptors are indeed known to spontaneously oscillate between two conformations, one active and the other inactive, in the absence of ligands^41–43^ (in the presence of these latter, the receptors are stabilized in an active state^44^). For GPCRs, this effect was already observed over 30 years ago with the delta opioid receptor^45^. Since then, and despite being often considered as noise, many more examples of such constitutive GPCR activity have been reported (with some receptors exhibiting high levels of agonist-independent activity such as the Ghrelin receptor^46^), as well as various diseases associated with GPCR mutations affecting this constitutive activity.

In the olfactory system, odorant-independent OSN basal activity has also been described in the context of axon guidance, where it plays an important role in regulating anterior-posterior targeting of OSN axons during development^47^. Constitutive OR activity thus appears to produce several different cellular states, whose number would be sufficient for defining enough non-overlapping neuronal categories to which distinctive rostro-caudal projection positions are assigned. The extent to which odor-induced activity experienced during development may influence the establishment of the glomerular map in addition to intrinsic activity, remains to be explored in details.

Finally, we identified the process at the origin of the agonist-induced variations of mRNA concentrations, which appears not to involve the half-life of mRNAs nor the nuclear export of these latter, but the modulation of transcription itself. Which molecular players are involved in this process is still unknown, but obvious candidates come to mind, such as the cAMP response element-binding protein (CREB), a transcription factor critical for activity-dependent neuronal plasticity^10^ that is involved in the activation-induced prolonged lifespan of OSNs^48^.

The mammalian olfactory mucosa thus represents a multifunctional sensor whose neuronal elements, that use olfactory receptors as internal and external chemical probes, are in constant evolution, adapting to the world via the activation of large-scale transcriptomic programs. Whether this transcriptomic diversity and dynamics functionally parallels the one recently observed in some central structures^49–52^, and whether the molecular tools involved in transcriptomic adjustments are shared between different circuits, remains to be explored. Given the transcriptomic diversity of neurons in the mammalian brain and knowing that 90% of non-sensory GPCRs are expressed in mammalian brains, the question is worth asking.

## Methods

### Animals

All experiments were conducted in accordance with the veterinary guidelines and regulations of the University and of the state of Geneva (Direction de l’expérimentation animale de l’UNIGE). C57BL/6J male mice were purchased at 5–7 weeks of age from Charles River Laboratories. Upon arrival, they were housed in groups of 4–5 animals. The following transgenic mouse lines were employed: *Olfr16-IRES-tau-GFP* (*Olfr16*^*tm2Mom*^, referred to as *Olfr16*^*GFP/GFP*^)*, Olfr151-IRES-tau-GFP* (*Olfr151*^*tm26Mom*^, referred to as *Olfr151*^*GFP/GFP*^)^53–55^. Transgenic mice were backcrossed on C57BL/6J background for over ten generations. Transgenic mice were bred and maintained at the University of Geneva. All mice were housed in standard type II cages with access to food and water ad libitum, on a dark/light cycle of 12/12 h, temperature between 21 and 22 °C, humidity between 45 and 55%.

### 10X single-cell RNA sequencing

#### Cell isolation, sorting, and sequencing

8 weeks old male C57BL/6J mice were used (*n* = 4). All experiments were performed during daytime. Mice were euthanized with intraperitoneal injection of pentobarbital (150 mg/kg) and their olfactory epithelia were immediately extracted and processed for tissue dissociation using the Papain Dissociation System (cat #LK003150; Worthington® Biochemical Corporation, New Jersey, USA) following the manufacturer’s protocol. Cell suspensions were then incubated with 2 μg/ml of Hoechst 33342 (a UV fluorescent adenine-thymine binding dye; #H1399, Life Technologies) at 37 °C for 15 min. Before fluorescence activated cell sorting (FACS) and to exclude dead cells, 1 μM of DRAQ7™ (a far-red fluorescent DNA intercalating dye; #DR71000, BioStatus) was added to the cell suspensions. Approximately 80,000 Hoechst+/DRAQ7− cells were collected from each sample, each in a final volume of 100 μl. After FACS sorting, cell suspensions were concentrated at 800 cells/μl. The targeted cell recovery was set to 10,000. In accordance with the Cell Suspension Volume Calculator Table of 10X Genomics, 22.6 μl of nuclease-free water was added to 20.6 μl of cell suspension and the samples were loaded on the 10X Genomics Chromium controller. GEM generation and barcoding, cDNA amplification and cDNA library construction were performed following the 10X Genomics Chromium Next GEM Single-Cell 3′ v3.1 protocol (dual index libraries). The cDNA libraries from each sample were then pooled and loaded at 2 nM on 2 lanes of the Illumina HiSeq 4000 system for paired-end sequencing.

#### scRNA-seq mapping and counting

fastq files were pre-processed with Cell Ranger version 6.0.1^56^ with default settings. Reads were mapped on the *Mus musculus* genome primary assembly reference ^38^ (GRCm38) using the STAR aligner^57^ implemented in Cell Ranger. A modified version of the Ensembl release 102 of the *Mus musculus* GTF annotation was used. This GTF file was updated with the re-annotation of the 3′UTR of olfactory receptor genes. The filtered feature-barcode matrices were used for downstream analysis. These matrices included a total of 21,809 cells (sample 1: 5364 cells; sample 2: 5696 cells; sample 3: 4756 cells; sample 4: 6004 cells).

#### scRNA-seq data filtering

Single-cell RNA sequencing data analyses were performed on R version 4.0.5 using the Seurat R package version 4.0.1^58,59^. Seurat’s functions were used with default settings unless specified. The standard analysis consisted of the following steps. First, the four 10X gene expression matrix files were individually loaded into R using the *Read10x* function of Seurat. The 10X data were then converted to Seurat objects using the *CreateSeuratObject* function of Seurat. The gene expression data was then normalized using the *SCTransform* function of Seurat^60^, and the top 5000 variable genes were determined for datasets integration^61,62^. Following the integration of the four datasets, a preliminary clustering was performed without any additional cell filtering in order to identify and remove cell clusters composed of blood, immune or suffering cells (i.e., cells exhibiting high expression levels of mitochondrial genes). PCA was performed on the integrated assay of the Seurat object using the *RunPCA* function of Seurat. A visual inspection of their explained standard deviation led to the selection of the top 9 PCs for subsequent cell clustering. To construct a shared nearest-neighbor graph, the above-mentioned PCs were used as input to the *FindNeighbors* function of Seurat (dims = 1:9). Cell clusters were then identified using the *FindClusters* function of Seurat with a clustering resolution of 1.1. This preliminary clustering yielded 28 cell clusters. Cluster-specific gene markers were then identified for cluster annotation. Briefly, the raw dataset containing cells sampled from all four mice was normalized by library size, scaled to 10^4^ and natural-log-transformed after adding a pseudocount of 1 using the *NormalizeData* function of Seurat. This normalized data was then used for differential expression analysis computed between each cell cluster and all other clusters taken together using the Wilcoxon rank sum test implemented in the *FindAllMarkers* function of Seurat (test.use = “wilcox”; only.pos = TRUE). Only genes with an adjusted *p* value below 0.05 were considered. Two blood cell clusters (*n* = 2) were identified based on their high expression of *Ptprc* and hemoglobin chain complex genes such as *Hba-a1* and *Hba-a2* (Supplementary Fig. 1). Immune cell clusters (*n* = 7) were identified based on their high expression of known immune cells markers such as *Igkc*, *Cd52*, *Cybb*, *Ctss*, *Tyrobp*, and *Gypa* (Supplementary Fig. 1). Suffering cell clusters (*n* = 4) were identified based on their high percentage of mitochondrial gene counts. This preliminary clustering led to the removal of 13 cell clusters from the dataset (*n* = 4708 cells). Furthermore, cells were also filtered out if their percentage of mitochondrial counts exceeded 10% of their total counts or if they expressed <1000 genes (*n* = 1242 cells). This preliminary analysis resulted in retaining 15,859 cells.

#### scRNA-seq clustering and analysis of main olfactory epithelium cells

The retained cells were used to identify cell clusters composing the mouse main olfactory epithelium (MOE). The corresponding datasets (one per mouse) were normalized and integrated, and cell clusters were identified as described in the previous paragraph (see *scRNA-seq data filtering*) with the following differences: the first 13 PCs were used for the *FindNeighbors* function of Seurat and a resolution of 0.3 was used for the *FindClusters* function of Seurat. This analysis led to the identification of 15 cell clusters. Cluster identities were then determined from the DEGs in each cluster (see above for more details). The markers described in Fletcher et al.^26^ were used for the annotation of the mouse MOE cell types. From the 15 clusters, 5 corresponded to mature OSNs based on their high expression of *Omp*, *Cnga2*, and *Gng13* but not *Gap43* (Supplementary Fig. 2a) These clusters were then merged together into only one cluster of mature OSN (Fig. 1b). To visualize the resulting 10 cell clusters on a 2-dimensional plot, the uniform manifold approximation and projection (UMAP)^63,64^ plot was computed using the *RunUMAP* function of Seurat and the first 13 PCs previously selected (dims = 1:13) (Fig. 1b).

Mature OSNs were selected from the main olfactory epithelium dataset for downstream analyses (*n* = 10,519 cells). For each mature OSN, the detected olfactory receptors were ordered based on their expression levels: 7025 OSNs displayed the expression of a single olfactory receptor, 2655 OSNs displayed the expression of two olfactory receptors and 684 OSNs displayed the expression of at least three olfactory receptors. To remove cells that could correspond to multiplets (among those co-expressing multiple olfactory receptors), the distribution of the expression levels of the highest expressed receptors was analyzed using the log-normalized data. An “is expressed” cutoff was set at three median absolute deviations from the median of the levels of expression of the highest expressed receptors. OSNs whose highest expressed receptor had an expression level below this cutoff were removed from the dataset (*n* = 229 cells). Moreover, OSNs that expressed more than one receptor at an expression level higher than this cutoff were also filtered out from the dataset (*n* = 344 cells). Finally, roughly 1.5% of the mature OSNs (*n* = 155 cells) did not show receptor expression and were also discarded from the dataset. The OSN population identity of each of the remaining cells (*n* = 9791 cells) was then determined based on the olfactory receptor that displayed the highest expression level in that given cell. This led to the identification of 952 OR-expressing OSN populations (*n* = 9741 cells) and 7 TAAR-expressing OSN populations (*n* = 44 cells), as well as a *Gucy1b2*-expressing OSN population (*n* = 6 cells). OSN populations represented by at least 3 cells in the dataset were included for clustering and downstream analyses (*n* = 9539 cells).

Two parallel analyses were carried out: by keeping or removing the olfactory receptor genes from the count matrix. The corresponding datasets were normalized and integrated, and cell clusters were identified as described above (see *scRNA-seq data filtering*), but with a substantial modification in the way PCs were chosen for downstream analyses. Rather than selecting the PCs by relying on the visual inspection of their explained standard deviation, the *KneeLocator* function of the kneed python package version 0.7.0^65^ was used with the following parameters: S = 1, curve = “convex”, direction = “decreasing”. This algorithm was applied on the explained standard deviation of the top 50 PCs to detect the elbow in the decrease of the explained standard deviation of the successive PCs (Supplementary Fig. 3a, b, d, e). The location of the elbow was then used as a threshold to retain the top relevant PCs for downstream processing of the datasets. The analyses described in *scRNA-seq data filtering* were thus carried out with the following differences: the percentage of mitochondrial gene counts were used as confounder variables in the *SCTransform* function of Seurat (vars.to.regress = “percent.mt”); the first 14 or 15 PCs (explaining 47% and 48% of the variance calculated with the top 50 PCs, respectively) were used for the *FindNeighbors* and *RunUMAP* functions of Seurat for the analyses including or not the olfactory receptor genes, respectively; and a resolution of 2.1 or 1.6 was used for the *FindClusters* function of Seurat for the analyses including or not the olfactory receptor genes, respectively. These concurrent analyses led to the identification of 29 (including olfactory receptor genes) or 23 (not including olfactory receptor genes) cell clusters, respectively. Cluster identities were then determined from the DEGs in each cluster (see *scRNA-seq data filtering* for more details). After cluster merging, a total of 10 clusters were retained, which were then subdivided into groups of “dorsal” or “ventral” clusters based on their complementary expression of *Nqo1* (a dorsal mature OSN gene marker) or *Nfix* (a ventral mature OSN gene marker), respectively. These broad clusters were each composed of five subclusters characterized by their expression of specific markers genes or absence of them: *Dlg2*+, *Calb2*+, *Cd55*+, *Cd36*+ and *Dlg2*−; *Calb2*−; *Cd55*−; *Cd36*– clusters. The clustering similarity between the two analyses (i.e., including or excluding the olfactory receptor genes) was computed with the normalized mutual information metric using the *compare* function of the igraph R package version 1.2.6 (method = “nmi”).

Similar to what was performed per cluster, OSN population-specific gene markers were identified using the log-normalized UMI counts and the *FindAllMarkers* function of Seurat (test.use = “wilcox”; only.pos = TRUE). In Fig. 1g, the largest OSN population from each cluster was selected and the gene expression levels of its cells were compared to those of all other cells from the dataset. In Fig. 1h, the six largest OSN populations from the *Dlg2-*, *Calb2-*, *Cd36−*, *Cd55*− ventral cluster were selected and for each of these populations the gene expression levels of their cells were compared to those of all other cells from that specific cluster. Only genes expressed in at least 70% of the cells of the given population and that yielded an adjusted *p* value below 0.05 were considered. For plotting, the log-normalized data was scaled and centered using the *ScaleData* function of Seurat, and the extreme values were clipped and set to the lower and upper limit values of the 95% confidence interval of the data using the *clip.data* function of the fsbrain R package version 0.4.3 (lower = 0.025; upper = 0.975)^66^.

### Transcriptomic, genomic, and amino acid distances

#### Transcriptomic Euclidean distance calculation between pairs of OSNs

To test whether OSN populations (defined by the OR gene they express) are transcriptionally dissimilar from each other, the pairwise transcriptomic Euclidean distances between pairs of OSNs were computed on the first 15 PCs of the mature OSN dataset devoid of OR gene counts (computed from the sctransform-normalized and integrated count matrix; see *scRNA-seq data filtering* for more details). The Euclidean distances were calculated on the PC space used for clustering and UMAP representation, rather than the gene counts, to faithfully represent and quantify the co-clustering of OSNs expressing the same receptor observed in Fig. 1d. Note that when the analysis was performed on the mature OSN dataset including the OR gene counts, the top 14 PCs were used (the right part of Fig. 1e; with OR genes).

In Fig. 1e and Supplementary Figs. 5–7, pairwise Euclidean distances were calculated between pairs of OSNs pertaining to populations represented by at least 3 cells in the dataset. These distances were sorted into two categories: distances between pairs of OSNs expressing the same olfactory receptor (intra) or pairs expressing different receptors (inter). To compare and measure the dissimilarity between these two distributions of pairwise transcriptomic Euclidean distances, the Cohen’s d was calculated between the intra and inter distributions (Fig. 1e) using the *cohen.d* function of the effectsize R package version 0.8.1 with default settings^67^. Moreover, to test if the difference in the distributions of Euclidean distances between pairs of OSNs expressing the same or different olfactory receptors was not a random effect, we generated a permuted dataset by randomly redistributing the cell identities (and hence the corresponding OR identities) of the top 15 PCs and recalculated pairwise distances for OSNs with identical random identities (intra perm). By iterating this process, the random distribution was estimated from 1000 permuted datasets. As described above, the Cohen’s d was then calculated between the intra or inter OSN population transcriptomic Euclidean distance distributions and each of the permuted distributions. This resulted in 1000 Cohen’s d values for each set of comparisons (i.e., intra versus intra perm and inter versus intra perm). The two distributions of Cohen’s d values were then compared using the two-sample Kolmogorov–Smirnov test. When this analysis was performed on each cluster separately, the original PC space was used for distance calculation but only the distances between cells from that given cluster were retained for the analysis, and the *p* values of the two-sample Kolmogorov–Smirnov test were adjusted for multiple comparisons using the Bonferroni correction method, as indicated in the figure legends.

#### Evaluation of the effect of PC choice on transcriptomic Euclidean distances between pairs of OSNs

To evaluate the effect of the choice of PCs on the results obtained, the Cohen’s d values were calculated to measure the dissimilarity between the distributions of intra and inter OSN population pairwise transcriptomic Euclidean distances, as described in the previous paragraph, when varying the number of top PCs used for the analysis (Supplementary Fig. 3c, f). We found that the discriminability between the intra and inter OSN population transcriptomic Euclidean distances reaches a plateau with the top 11 PCs (or higher), and the computational approach to select the top PCs for downstream analysis is robust and allows to capture most of the OSN population-based variance in the dataset.

#### Evaluation of the effect of dataset integration on transcriptomic Euclidean distances between pairs of OSNs

Though dataset integration corrects for batch effects found in the data, it also alters the gene expression profile of the cells. When dataset integration is performed, shared cell types are identified between datasets and their gene expression profiles are “corrected” (i.e., altered) so to remove batch effects that might hamper their co-clustering during downstream analysis. Given the differences in the frequencies of OSN populations, dataset integration might impact differently small and large OSN populations. For the large OSN populations, whose cells were sampled from most if not all mice, dataset integration might only correct for batch effects found between the individual datasets, as the search for common cell types (i.e., anchors) between the datasets might result in the identification of cells expressing the same OR. This is not the case for small OSN populations whose cells are sampled from a few of the mice used in this experiment. For example, an OSN population formed of 3 cells can be sampled from only one mouse, and therefore none of the datasets from the other mice would contain cells from that given population. A byproduct of this could be the alteration of the transcriptomic profiles of two small OSN populations so to make them more similar for dataset integration, especially since the OR gene expression information was removed from the dataset, which could have helped to discriminate these two populations. In other words, the neighbors of cells from a given small OSN population sampled from a given mouse can be identified as the cells pertaining to another small OSN population sampled from another mouse. Hence, in this case, the search for neighbors between the datasets can be biased towards small OSN populations. To evaluate this, OSN populations were binned based on their size into equal frequency bins and the two extreme bins (one formed by populations of small sizes, and the other of large sizes) were selected for further analysis (Supplementary Fig. 7a). Subsequently, the inter OSN population pairwise transcriptomic distances between pairs of OSNs from each of the two bins were subsampled to include the distances between 1/3 of the OSNs from each population, and the subsampled distances were compared to each other and to all intra OSN population distances using Cohen’s d as described above (see *Transcriptomic Euclidean distance calculation between pairs of OSNs*). This process was repeated 10,000 times, hence resulting in 10,000 Cohen’s d values for each set of comparisons (i.e., inter small versus inter large, inter small versus intra, and inter large versus intra) (Supplementary Fig. 7b, c).

In parallel, the analysis detailed in *Transcriptomic Euclidean distance calculation between pairs of OSNs* was also performed for each mouse without dataset integration (Supplementary Fig. 6). Following PC selection using the kneed python package described above, the analysis was computed on the top 14 PCs for mouse 1, the top 12 PCs for mouse 2, the top 10 PCs for mouse 3, and the top 14 PCs for mouse 4.

#### Transcriptomic Euclidean distance calculation between OSN populations

To compare the transcriptomic Euclidean distances between OSN populations with the genomic or phylogenetic distances between the olfactory receptors they express, population transcriptomic distances were calculated on the same PC space used for clustering and UMAP representation (i.e., top 15 PCs of the mature OSN dataset without OR gene counts). To this end, OSN population centroids were estimated on the PC space and the distances between the centroids were used as a proxy for the distances between pairs of OSN populations (Fig. 2e–g and Supplementary Fig. 8d). To reduce small sample biases in centroid estimations, this analysis was restricted to OSN populations represented by at least 10 cells in the dataset.

#### Functional OR gene identification and OR phylogeny

The functional OR phylogeny was partly built from the same sequence set as used in von der Weid et al.^25^. To constitute this set, OR coding sequences were identified de novo in the mouse genome assembly GRCm38 using TBLASTN searches with previously annotated mouse OR protein sequences as queries. The hits were manually curated to filter out putative non-functional receptors. The criteria to consider an OR to be functional was the conservation of evolutionary constrained residues^68^, the integrity of the seven transmembrane domains and the absence of intron within the coding sequence^69^, resulting in a set of 1141 putatively functional OR. After this filtering, 11 filtered out ORs were retrieved as they were found to be expressed in a monoallelic fashion in one or more OSNs, in our scRNA-seq data. For these ORs, we used coding sequences as annotated in Ensembl version 102. Notably, 8 of these 11 ORs have their coding sequence spanning two exons, with most of the coding sequence (covering the seven transmembrane domains) included in the last exon.

A multiple sequence alignment including the resulting OR protein sequence set was obtained with Clustal Omega v1.2.4^70^, using the*—full* and—*full-iter* options. The resulting alignment was trimmed to keep the sites between the most conserved start methionines and the last position with less than 90% of gaps.

The maximum likelihood phylogeny of the mouse functional ORs was calculated with Phyml version 20120412^71^ using the following parameters: -d aa -m JTT -f e -v e -c 4 -a e -s BEST -o tlr. The resulting tree was rooted on the node at the origin of class I and class II ORs. For display of transcriptomic identities of associated OR in Fig. 2a, each OSN population was assigned to the transcriptomic cluster to which the majority of cells belong. In case of equivalences, we assigned the transcriptomic cluster randomly. Branch tips were colored according to the assigned transcriptome cluster identity of OSNs expressing the corresponding OR.

#### Genomic distances and gene cluster definition

Pairwise genomic distance between OR genes was measured as the distance in base pairs between start codons of OR genes. For the 8 OR genes that have their start codon on another exon, we instead used the first position of the last coding exon. Genomic distances were only obtained between genes in the same chromosome.

Pairwise distances between adjacent genes were used to aggregate OR genes in cluster. For this, the sorted distances were split into two groups using the Jenks natural break optimization for *k* = 3. In that manner, the middle break is used to separate unbiasedly two categories of distances: the smaller distances representing the intracluster distances and the longer distances representing the intercluster distances. Next, we calculated the mean and the standard deviation of the intracluster distances and defined the clustering threshold as the mean plus 3 times the standard deviation. Finally, gene clusters were obtained by aggregating neighboring genes whose genomic distance was closer to each other than the clustering threshold.

#### Amino acid difference metric

Pairwise amino acid difference was measured on the protein alignment that was used for the phylogenetic reconstruction. For a given pair of aligned sequences, each substitution was scored according to the Miyata amino acid replacement matrix^72^. Insertions were scored as the mean replacement scores of each additional amino acid. The sum of these scores gave the pairwise amino acid difference.

#### Evaluation of genomic proximity and sequence identity as determinants for transcriptome identity

In Fig. 2f, we defined thresholds of genomic distance and amino acid difference to attribute pairs of OSN populations as being close in terms of genomic proximity between the OR genes they express or in terms of sequence identity between their respective OR. For genomic proximity, we evaluated all intergenic distances between neighboring ORs belonging to the same cluster and chose the 95th percentile of this distribution as the threshold value for a pair to be considered close. For sequence identity, we evaluated all pairwise amino acid differences between ORs belonging to the same class and chose the 5th percentile of this distribution as the threshold value for a pair to be considered close. For Fig. 2g, a pair was considered distant in terms of genomic proximity when the corresponding OR genes were located in different OR gene cluster. A pair was considered distant in terms of sequence identity when the amino acid difference between their corresponding ORs was higher than the threshold used to identify the close pairs.

### Chemicals

Odorants were directly purchased from Sigma-Aldrich, ethyl isobutyrate (W242802), acetophenone (42163). Lyral was obtained as a generous gift from Dr. Christian Margot (Firmenich).

### Odorant exposure

For all odorant exposures, on the day preceding the odorant exposure mice were isolated and single-housed in a standard type II long cage. Exposure assays started at 8:00 a.m. and lasted 5 h. For the exposed condition, a cotton swab was imbibed with 200 µL of 5% odorant in a DMSO solution and was placed in the cage, while for the control condition, a cotton swab was imbibed with 200 µL of DMSO only and was placed in the cage.

### FACS-seq

#### Cell isolation, sorting, and sequencing

7 weeks old *Olfr16*^*GFP/GFP*^ and *Olfr151*^*GFP/GFP*^ male mice were used to isolate single fluorescent OSN populations from the whole MOE. Mice were exposed as described above to lyral and acetophenone, respectively. Control mice from each transgenic line were exposed to DMSO only. For each condition, there were 3 samples, where each sample was constituted by a pool of 4 mice. After odorant exposure, mice were euthanized by intraperitoneal injection of pentobarbital (150 mg/kg), the whole MOE was extracted and OSNs were dissociated by adapting the protocol described in Kaur et al.^73^. Briefly, the collected epithelia were minced inside a tube containing a dissociation buffer (D-csyteine-HCl 1 M, EDTA 100 mM, Papain 0.3U/µL, DNAse I (Ambion) 2 U/µL and DNAse I 10× buffer (Ambion), dissolved in freshly prepared and oxygenated cold aCSF). The aCSF composition was the following: 118 mM NaCl, 25 mM NahCO3, 10 mM D-glucose, 2 mM KCl, 2 mM MgCl2, 1.2 mM NaH2PO4, 2 mM CaCl2. Samples were then placed at 37 °C for a total of 25 min allowing enzymatic dissociation of the tissues, during which they were subjected to a trituration step every 5 min using polished glass pipettes. At the end of the dissociation, each sample was filtered through a 20 µm Nylon filter (Falcon), and centrifuged for 5 min at 200G. The supernatant was discarded and replaced with ice-cold aCSF. Before FAC-sorting, samples were incubated at 37 °C for 20 min with Hoechst 33,342 (1 mg/mL) to label live cells. Cell-sorting was performed on an AriaII (BD Biosciences) cell-sorter, gated on Hoechst and GFP fluorescence. Cells were collected directly in lysis buffer from the Qiagen RNeasy plus micro kit. For the *Olfr16*^*GFP/GFP*^ mice, 100 cells were collected per individual, resulting in 400 cells per biological pool. For the *Olfr151*^*GFP/GFP*^ mice, 50 cells were collected per individual, resulting in 200 cells per biological pool. The difference in the total number of cells collected per experiment derives from the original respective OSN population sizes in the epithelium^74^. The RNA extraction was performed according to the Qiagen RNeasy plus micro kit protocol. The SMARTer™ Ultra Low RNA kit from Clontech was used for reverse transcription and cDNA amplification (12 PCR cycles) according to the manufacturer’s specifications, starting with a total volume of 9.5 µL per sample as total RNA input. 200 pg of cDNA were used for library preparation using the Nextera XT kit from Illumina. Library molarity and quality was assessed with the Qubit and Tapestation using a DNA High sensitivity chip (Agilent Technologies). Libraries were pooled at equimolarity and loaded at 11 pM for clustering on a Single-read Illumina Flow cell for the *Olfr16*^*GFP/GFP*^ experiment. Reads of 50 bases were generated using the SBS HS v3 chemistry on an Illumina HiSeq 2500 sequencer. Deep sequencing of the Olfr16 dataset yielded a mean of 37.2 M (±1.3 M) short single-reads for the control condition, and a mean of 35.4 M (±4 M) short single-reads for the exposed condition. For the Olfr151 experiment, libraries were loaded at 2 nM for clustering on an Illumina HiSeq 4000 sequencer. Deep sequencing of the Olfr151 dataset yielded a mean of 58.5 M (±1.5 M) short single-reads for the control condition, and a mean of 62.1 M (±1.3 M) short single-reads for the exposed condition.

#### FACS-seq mapping and counting

STAR (v.2.7.0)^57^ was used to map the generated reads on the Ensembl *Mus musculus* genome primary assembly ref. ^38^ (GRCm38) that included the IRES-tau-GFP sequence. Gene expression quantification was carried out using featureCounts version 1.6.3^75^.

#### FACS-seq data filtering

To filter out lowly- and non-expressed genes for each OSN population (Olfr16 and Olfr151), a count threshold was determined to exclude all genes with expression values below this threshold across either the 3 control or 3 exposed samples. Briefly, the density distribution of gene counts was used to calculate the local minimum and this value was set as the threshold.

#### FACS-seq gene expression analysis

The DESeq2 package (v.1.30.1) was then used to perform differential expression analysis. After fitting a negative binomial generalized linear model (GLM), the Wald test (two-tailed) was used to test for significance of gene expression at a log_2_ fold-change threshold of 0.5. To control the false discovery rate, the Wald test *p* values were adjusted for multiple comparisons using the Benjamini–Hochberg procedure^76^. Fold changes of DEGs were estimated using the apeglm R package version 1.16.0.

#### Gene Ontology enrichment analyses

All GO enrichment analyses were performed testing GO terms mapped to the DEGs common to both analyzed OSN populations (Olfr16 and Olfr151) against a background of GO terms mapped to all other genes commonly expressed in both OSN populations. DEGs were analyzed for GO enrichment by the topGO package using the runTest function with the “classic” algorithm and the Fischer statistics. To control the false discovery rate, the *p* values were adjusted for multiple comparisons using the Benjamini–Hochberg procedure^76^. The result of the GO terms analysis were then plotted with the ggplot2 function in R.

### Bulk RNA-seq

The raw data generated in this experiment was previously published in von der Weid et al.^25^.

#### Bulk RNA-seq mapping and counting

The mapping and counting of bulk RNA-seq data was performed exactly as described for FACS-seq data above (*FACS-seq mapping and counting*, *data filtering* and *expression analysis*).

### Exon-intron split analysis

Using the STAR read aligner tool (v.2.7.0)^57^, reads from the FACS-seq and 8 weeks old male RNA-seq experiments were mapped to the *Mus musculus* Ensembl transcriptome reference (GRCm38 from Ensembl). The annotation file used for this analysis only contained protein coding gene annotations. Gene expression quantification was carried out using featureCounts^75^ version 1.6.3. To quantify intronic reads for a specific gene, we subtracted the reads mapped on the exon from the reads mapped to the entire transcript. For data shown in Fig. 5c, d, f, g, for each gene and feature counted (i.e., exonic or intronic reads), the DESeq2 normalized counts of the exposed samples were normalized by the mean DESeq2 normalized counts of the control samples. Hence, each dot on the plot corresponds to an odorant-exposed sample. The values on the y-axis are raw fold-change values, but the scale of the y-axis is in log 2. For Fig. 5i, given the low amount of intronic reads in OR genes, we only show OR genes with more than 14 intronic read counts. To this list, we added *Olfr171*, as it was described in Fig. 5j. Transcriptional downregulation of intronic and exonic features in exposed mice was tested by a two-sample independent *t* test using a linear model in R. P values were adjusted using the FDR method. To represent the raw data of the exon-intron split analysis prior to fold-change calculation (shown in Supplementary Fig. 12), we centered and scaled (i.e., z-scored) the DESeq2 normalized counts of all samples per gene and features counted (i.e., exonic or intronic reads).

### In situ hybridization

C57BL/6 J adult male mice were exposed to odorants for 1 h (four mice exposed to ethyl isobutyrate, four mice exposed to DMSO only), after which they were euthanized. Heads were placed in 10% formalin, purged of gas, left overnight at 4 °C, transferred to 15% sucrose for 12 h, followed by 30% sucrose for 12 h. They were embedded in OCT and frozen. The main olfactory epithelium was cut in 16–18 μm coronal sections with a cryostat-microtome. Slides were conserved at −80 °C until use. RNA probes were designed to have a maximum identity with aspecific targets of 80% over a 100-bp window. Primers to amplify the probe for *Olfr171* were: AGTGCCTTCTCTTGGCAGT (forward) and GAGTGTGGGTGTCAGGATGG (reverse). The probe was transcribed with fluorescein-labeled UTP using the Roche RNA Labeling Kit and In-Vitro Transcription Kit (Roche, ref. 11175025910) following the manufacturer’s protocol. Slides were post-fixed in 10% formalin for 15 min, and washed for 3 min in PBS. Slides were incubated in 0.1% H_2_O_2_ for 30 min and then washed twice in PBS for 3 min. Slides were then treated with 10 μg/ml proteinase K in TE for 5 min, followed by an incubation in 10% formalin for 10 min and washed in PBS for 3 min. 0.2 M HCl was then added to the slides for 10 min, followed by a 3 min PBS wash. Then the slides were pre-incubated in 0.1 M triethanolamine HCl, pH 8 for 1 min and incubated in 0.1 M triethanolamine HCl with acetic anhydride for 10 min, followed by a 3 min PBS wash. Probes were denatured for 7 min at 70 °C and diluted 1:400 in 50% formamide, 10% dextran sulfate, 1 μg/μl tRNA and 1× Denhardt’s solution in nuclease-free water. Slides were incubated in the hybridization buffer for 14–18 h at 65 °C. Slides were washed 2 × 30 min at 65 °C and 1 × 30 min at 20–25 °C in 1× SSC, 50% formamide, 0.1% Tween-20, H2O DEPC. They were then pre-incubated 30 min with 1× MABT with 2% Blocking Reagent (Roche, ref. 001,11,096,176). Roche Anti-Fluorescein POD Fab fragments (Roche, ref. 11426346910) was diluted 1:200 in pre-incubation mix and slides were covered with the antibody solution for 30 min. Slides were washed 3 × 5 min in TNT (150 mM NaCl, 100 mM Tris, HCl to pH 7.5 in 10 L, 0.05% Tween-20), treated with PerkinElmer Biotinylated Tyramide 1:50 in Amplification Diluent for 30 min, and washed 3 × 5 min in TNT. Finally they were treated with Alexa-488-labeled Streptavidin (Life Technologies) 1:100 in PerkinElmer Amplification Diluent for 30 min, washed 3 × 5 min in TNT and then incubated with PBS. Fluorescence intensity was assessed by measuring the total fluorescence of a disk with an area of 1.8 μm^2^ in diameter that comprised the transcription foci using the ImageJ software. Fluorescence intensity ratios were calculated by dividing the fluorescence level of each exposed neuron to the mean fluorescence of control neurons that had been processed in parallel.

### RNAscope in situ hybridization

5-weeks-old male and female *Olfr16*^*GFP/GFP*^ mice were exposed to odorants for 5 h (*n* = 3 mice exposed to 5% lyral in DMSO, *n* = 3 mice exposed to 5% acetophenone in DMSO and *n* = 6 mice exposed to DMSO only). Exposure to the two odorants took place on different days, each time with a control group. Lyral is a known ligand for Olfr16, while acetophenone is known not to activate this receptor^25^. Mice were euthanized and heads were fixed in freshly prepared 4% PFA overnight at 4 °C. Tissue was sequentially immersed in 10% sucrose for 18 h, 20% sucrose for 8 h and 30% sucrose for 8 h, always at 4 °C. Heads were embedded on OCT, frozen in liquid nitrogen and stored at −80 °C until sectioning. The main olfactory epithelium was cut on a cryostat microtome in 16 μm coronal sections. For each mouse, 2 sections were used for RNAscope ISH. Endogenous GFP signal was used to select section with a high number of OSNs expressing *Olfr16*. RNAscope staining was performed according to the manufacturer’s protocol (RNAscope™ Multiplex Fluorescent V2 Assay, ref. 323136, Advanced Cell Diagnostics). Pretreatment was performed according to guidelines for fixed-frozen tissue and included post-fixation, de-hydration, hydrogen peroxide treatment, 5 min target retrieval and 5 min Protease III treatment. Sections were labeled with probes for *Mustn1* and *Olfr16* (Mm-Mustn1, ref. 568751, Mm-Olfr16-C3, ref. 538841-C3, both from Advanced Cell Diagnostics). Probes were visualized with Opal fluorophores (OpalTM 570, ref. FP1488001KT, OpalTM 690, ref. FP1497001KT, Akoya biosciences). Sections were counterstained with DAPI and mounted with ProLongTM Gold antifade (ref. P36935, Invitrogen).

Slides were imaged with a Zeiss LSM800 confocal microscope equipped with 405, 555, and 639 nm laserlines, using a 40 × 1.3 NA oil-immersion objective. Cells of interest were identified based on the expression of Olfr16. Images of 5 Z stacks were acquired and exported as orthogonal projections for analysis. Images were analyzed using the CellProfiler software, version 4.2.1^77^. with the Speckle Counting pipeline. As the cell-density within the main olfactory epithelium does not allow for automated cell segmentation, the contour of *Olfr16*-positive cells was manually drawn based on the *Olfr16*-labeling and DAPI staining (IdentifyObjectsManually). Then, the number of *Mustn1*-positive puncta were counted automatically within this ROI. The same settings for probe detection were used for all images pertaining to the same experiment, including both exposed and control mice. As *Olfr16* is a highly expressed gene (always > 15 puncta/cell) the number of fluorescent spots per cell can not be quantified^78^, we recorded the total number of labeled pixels and the total intensity of pixels within each *Olfr16*-positive cell. These are arbitrary units and do not reflect absolute transcription values, but allow us to estimate changes in transcription between control and exposed mice.

### Statistics and reproducibility

Statistical tests and data representation were computed in R version 4. Data points were represented in the graphs when the number of dots was <10, otherwise the distribution of dots were represented in violin plots. All statistical tests were performed considering a two-sided alternative hypothesis. Continuity correction was applied to Wilcoxon rank sum tests. Significance was assessed for *p* values < 0.05. Detail of all statistical tests are shown in Supplementary Table 1.

For transcriptional quantification by RNAscope, the experiment was performed once for each odor and included non-exposed mice in both experiments. Each experiment included several independent biological samples from each condition. For transcriptional quantification by standard in situ hybridization, the experiments was repeated four times and included both control and exposed samples in each experiment.

### Reporting summary

Further information on research design is available in the Nature Research Reporting Summary linked to this article.

## Supplementary information

Supplementary Information

Reporting Summary

Description of Additional Supplementary Files

Supplementary Data 1

Supplementary Data 2

Supplementary Data 3

## Data Availability

The FACS-seq and single-cell RNA-seq data generated in this study have been deposited in the NCBI GEO database under accession code GSE185168. The two custom annotations of the mouse genome GRCm38 that were used for scRNA-seq, FACS-seq and bulk RNA-seq as well as the OR protein phylogeny have been deposited in figshare [10.6084/m9.figshare.c.5957625].  Source data are provided with this paper.

## Source data

Source Data

## Code availability

All original code used to analyze data reported in the paper are provided at the GitHub repository [https://github.com/irlabgenev/DREAM_pt2].
