# Publisher Correction: Transcriptional adaptation of olfactory sensory neurons to GPCR identity and activity

**DOI:** 10.1038/s41467-023-36849-7

**Published:** 2023-03-01

**Authors:** Luis Flores Horgue, Alexis Assens, Leon Fodoulian, Leonardo Marconi, Joël Tuberosa, Alexander Haider, Madlaina Boillat, Alan Carleton, Ivan Rodriguez

**Affiliations:** 1 Department of Genetics and Evolution, Faculty of Sciences, University of Geneva, Geneva, Switzerland; 2 Department of Basic Neurosciences, Faculty of Medicine, University of Geneva, Geneva, Switzerland

**Keywords:** Genetics of the nervous system, Sensory processing, Cellular neuroscience

Correction to: *Nature Communications* 10.1038/s41467-022-30511-4, published online 25 May 2022

The original version of this Article contained an error in Fig. 4j, in which the labelled GO terms ‘signaling’, ‘cell communication’ and ‘signal transduction’ were inadvertently shifted during production. The correct version of Fig. 4j is:
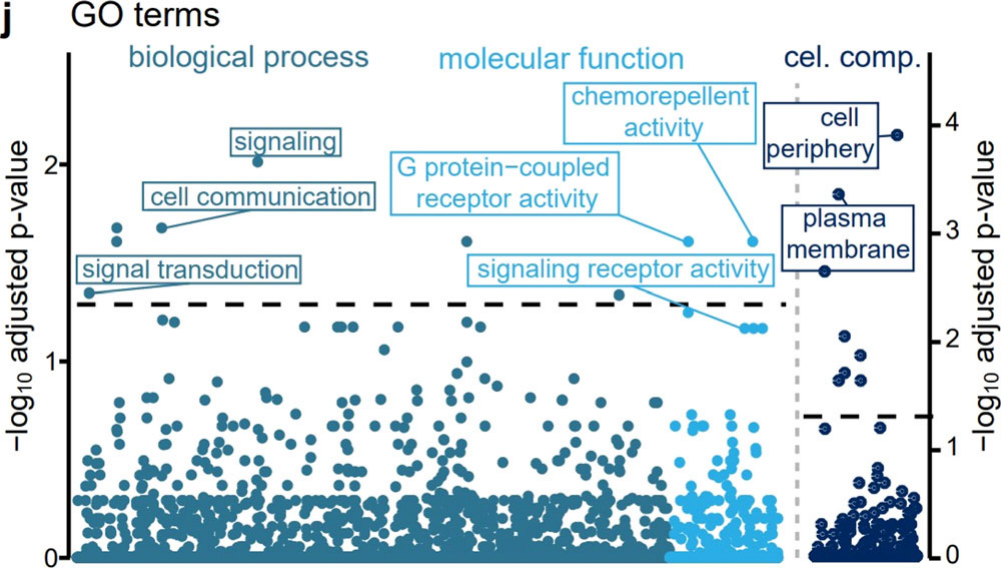


This has been corrected in both the PDF and HTML versions of the Article.

