# Supplementary material for: Transcriptional adaptation of olfactory sensory neurons to GPCR identity and activity
Source: Nat Commun. 2022 May 25;13:2929. doi: 10.1038/s41467-022-30511-4 (PMC9132991; doi:10.1038/s41467-022-30511-4)
Supplement: Supplementary file 1 — Supplementary Information [file 41467_2022_30511_MOESM1_ESM.pdf]

Supplementary Information

Transcriptional adaptation of olfactory sensory neurons to GPCR identity and activity

Luis Flores Horgue[1,+], Alexis Assens[1,+], Leon Fodoulian[1,2], Leonardo Marconi Archinto[1], Joël Tuberosa[1], Alexander Haider[1], Madlaina Boillat[1], Alan Carleton[2,*] and Ivan Rodriguez[1,*]

[1] Department of Genetics and Evolution, Faculty of Sciences, University of Geneva, Switzerland
[2] Department of Basic Neurosciences, Faculty of Medicine, University of Geneva
[+] equal contribution
[*] to whom correspondence should be addressed

Supplementary figures

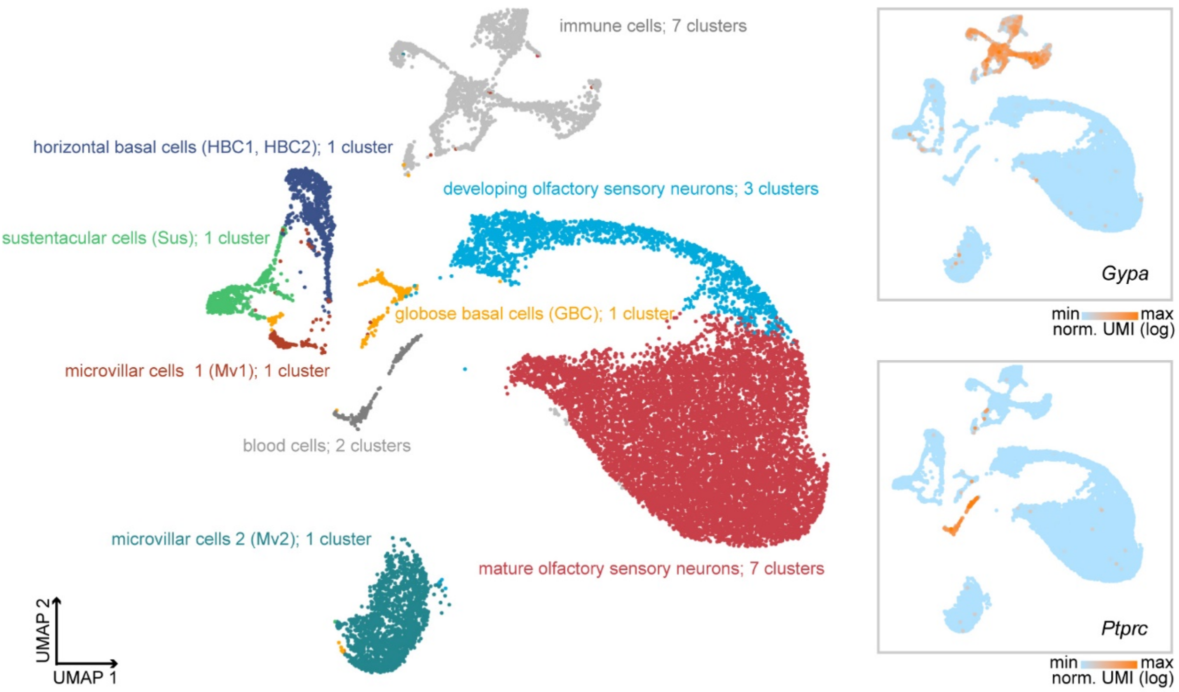

Supplementary Figure 1. Main olfactory epithelium clusters
Visualization of MOE cell clusters on a *UMAP* plot prior to filtering immune and blood cell clusters. Insets: normalized expression levels of immune (*Gypa*) and blood cell (*Ptprc)* gene markers.

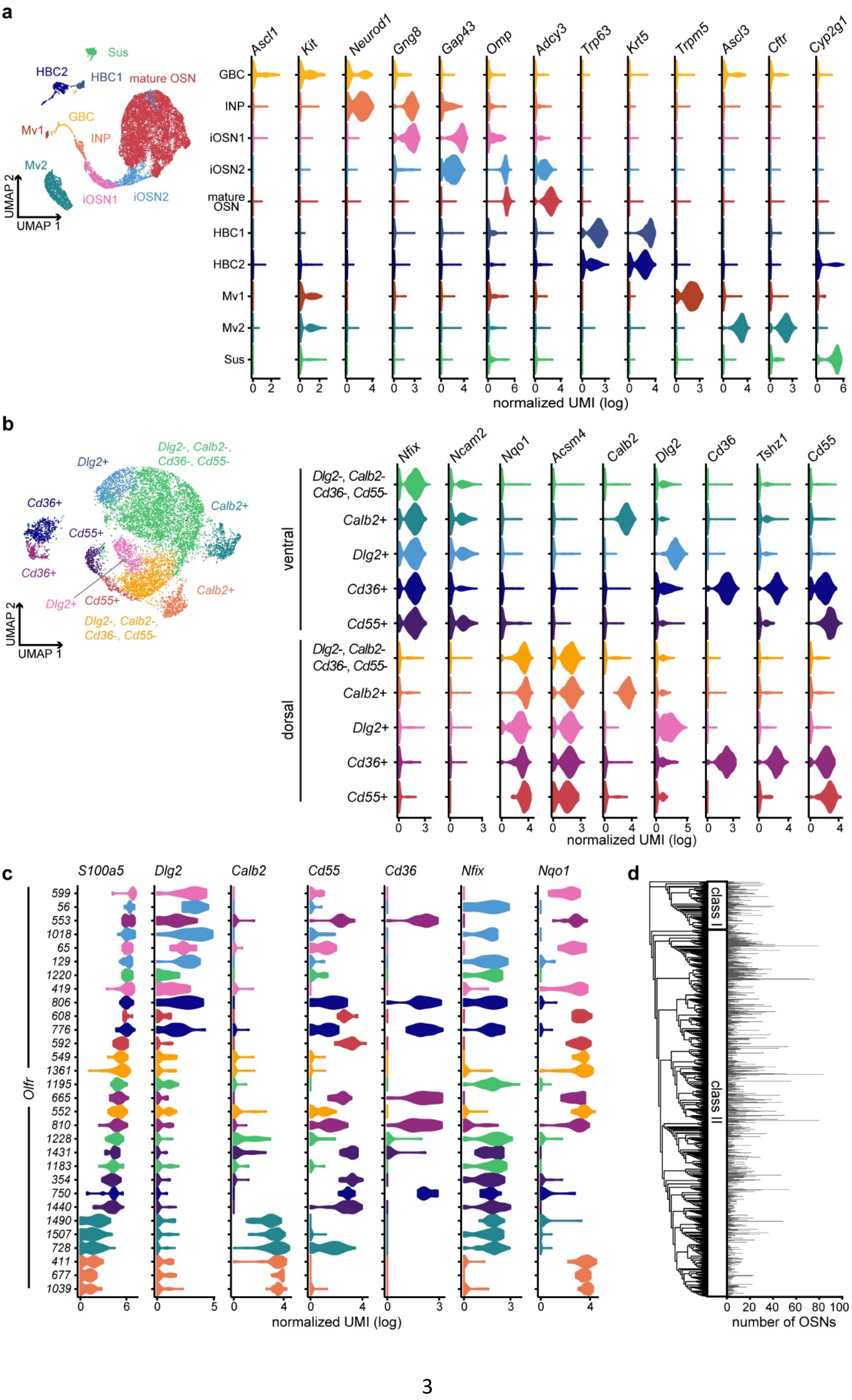
a
Sus
HBC2
HBC1
mature OSN
GBC
Mv1
INP
Mv2
iOSN1
iOSN2
UMAP 2
UMAP 1
Ascl1 Kit Neurod1 Gng8 Gap43 Omp Adcy3 Trp63 Krt5 Trpm5 Ascl3 Cftr Cyp2g1
GBC
INP
iOSN1
iOSN2
mature OSN
HBC1
HBC2
Mv1
Mv2
Sus
normalized UMI (log)
b
Dlg2+
Dlg2-, Calb2-, Cd36-, Cd55-
Cd36+
Calb2+
Cd55+
Cd36+
Dlg2+
Cd55+
Calb2+
Dlg2-, Calb2-, Cd36-, Cd55-
UMAP 2
UMAP 1
Nfix Ncam2 Nqo1 Acsm4 Calb2 Dlg2 Cd36 Tshz1 Cd55
ventral
Dlg2-, Calb2- Cd36-, Cd55-
Calb2+
Dlg2+
Cd36+
Cd55+
dorsal
Dlg2-, Calb2- Cd36-, Cd55-
Calb2+
Dlg2+
Cd36+
Cd55+
normalized UMI (log)
c
S100a5 Dlg2 Calb2 Cd55 Cd36 Nfix Nqo1
Olfr
599
56
553
1018
65
129
1220
419
806
608
776
592
549
1361
1195
665
552
810
1228
1431
1183
354
750
1440
1490
1507
728
411
677
1039
normalized UMI (log)
d
class I
class II
0 20 40 60 80 100
number of OSNs

Supplementary Figure 2. Cluster-specific markers

(**a**) Violin plots showing cluster-specific distribution of selective marker genes characteristic of the different cell types populating the nasal cavity (log normalized UMI).

(**b**) Violin plots showing cluster-specific distribution of selective marker genes characteristic of the identified mature olfactory sensory neuron (OSN) subclusters (log normalized UMI).

(**c**) Violin plots showing cluster-specific distribution of selective marker genes characteristic of the identified mature OSN subclusters (log normalized UMI) in various OSN populations. OSN populations are ordered by their mean expression of *S100a5*. The color of each violin plot indicates the cluster to which the majority of the cells from the given population pertain.

(**d**) Bar plot showing the number of cells per OSN population composed of at least 3 cells. Olfactory receptor genes are phylogenetically organized.

Source data are provided as a Source Data file.

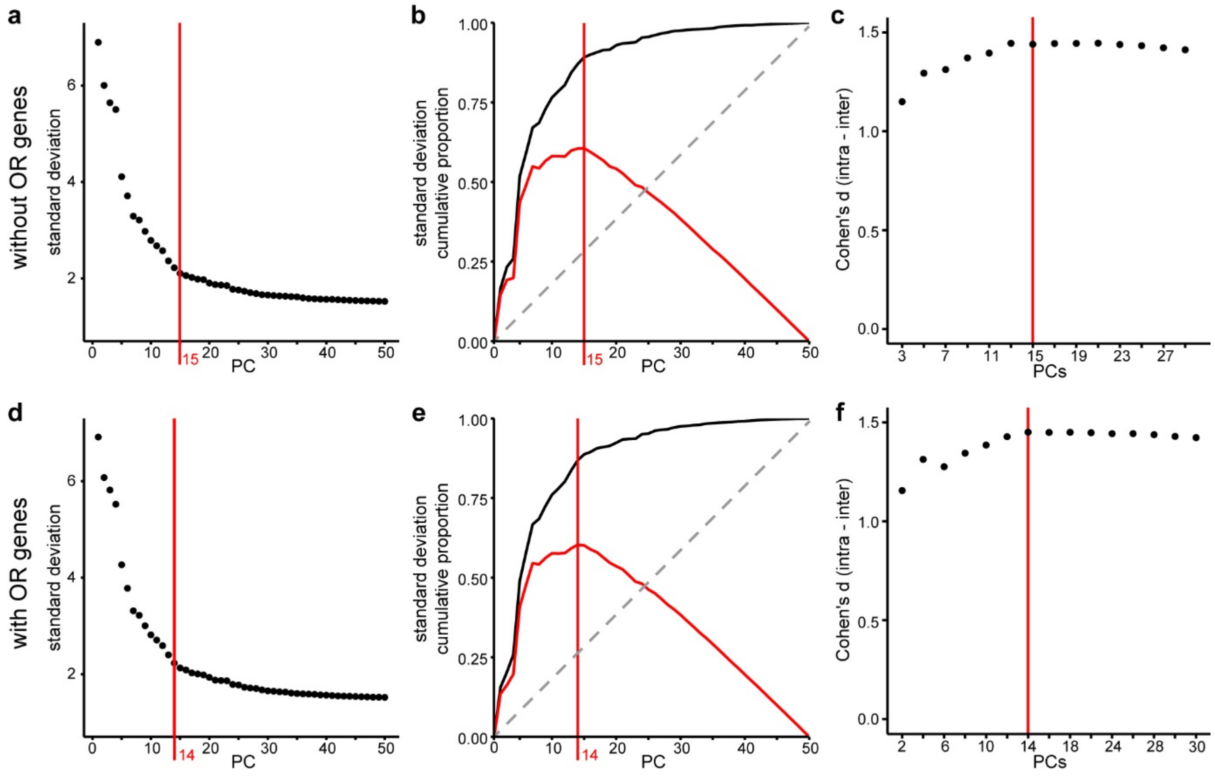

Supplementary Figure 3. Selection and evaluation of principal components (PCs) for downstream analysis

(**a**) Elbow plot showing the decrease in standard deviation of consecutive PCs computed on the OSN dataset without the inclusion of odorant receptor (OR) gene counts. The red vertical lines correspond to the PC at which the elbow in the decrease in standard deviation is located.

(**b**) Plot showing the cumulative proportions of the standard deviations of the first 50 consecutive PCs (black line) from (**a**). The red line corresponds to the difference between the black line and the dotted grey line (i.e. diagonal, representing the case if all PCs had the same standard deviation). The vertical red line corresponds to the knee/elbow in the decrease in standard deviation of consecutive PCs.

(**c**) Dot plot showing the variation in the Cohen's d values computed between the distributions of within (intra) and between (inter) OSN population pairwise transcriptomic distances when varying the number of PCs used for the analysis excluding OR gene counts.(**d-f**) Same analyses than in (**a-c**), but for the dataset including OR gene counts.

Source data are provided as a Source Data file.

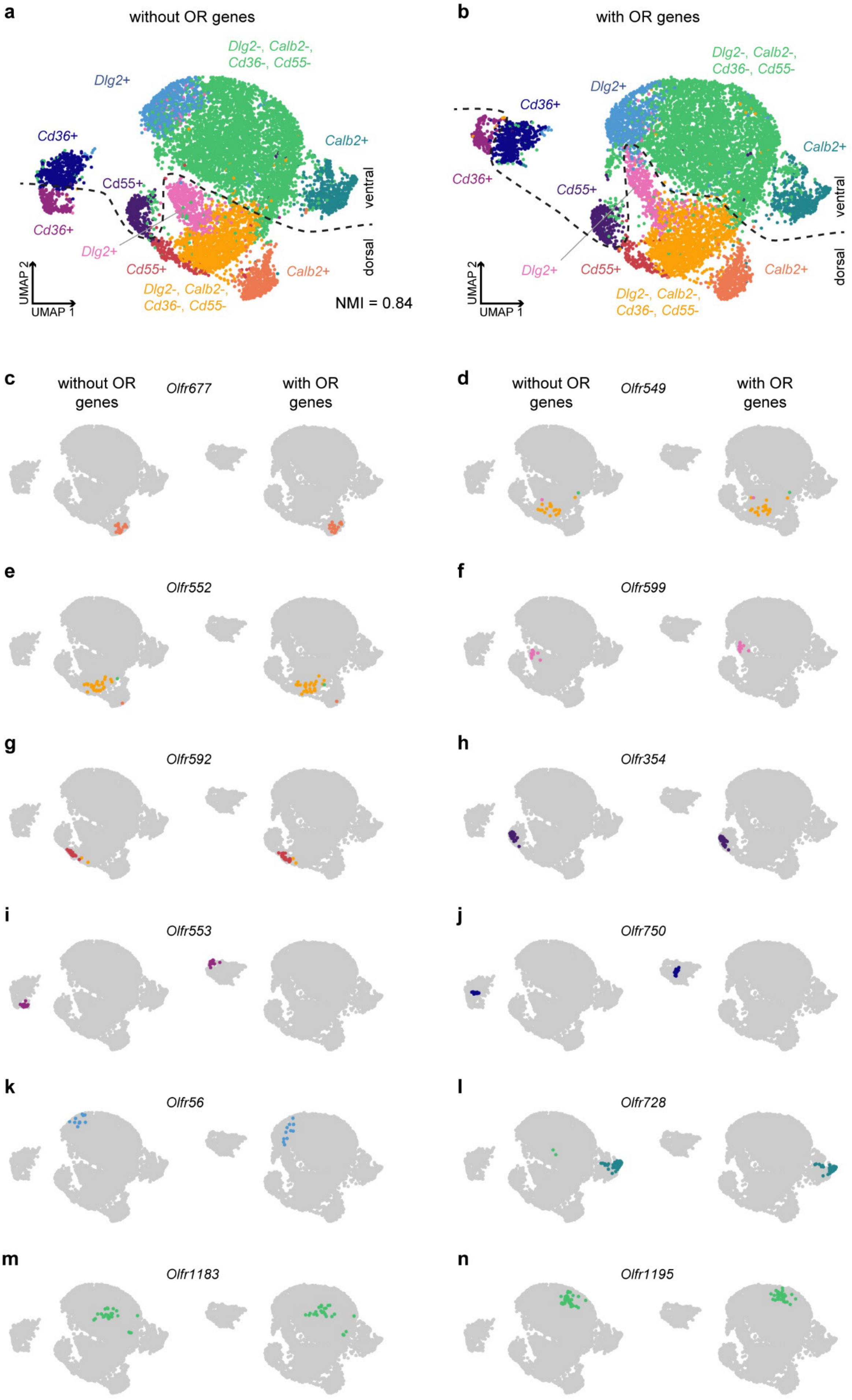
a
without OR genes
Dlg2-, Calb2-, Cd36-, Cd55-
Dlg2+
Cd36+
Calb2+
Cd55+
ventral
Cd36+
Dlg2+
Cd55+
Calb2+
dorsal
UMAP 2
UMAP 1
Dlg2-, Calb2-, Cd36-, Cd55-
NMI = 0.84
b
with OR genes
Dlg2-, Calb2-, Cd36-, Cd55-
Dlg2+
Cd36+
Calb2+
Cd36+
Cd55+
ventral
Dlg2+
Cd55+
Calb2+
dorsal
UMAP 2
UMAP 1
Dlg2-, Calb2-, Cd36-, Cd55-
c
without OR genes
Olfr677
with OR genes
d
without OR genes
Olfr549
with OR genes
e
Olfr552
f
Olfr599
g
Olfr592
h
Olfr354
i
Olfr553
j
Olfr750
k
Olfr56
l
Olfr728
m
Olfr1183
n
Olfr1195

Supplementary Figure 4. Clustering of olfactory sensory neuron populations without odorant receptor data

(**a**-**b**) Visualization of MOE cell clusters on a *UMAP plot* computed (**a**) with or (**b**) without the inclusion of olfactory receptor genes in the count dataset. The normalized mutual information (NMI) score indicates the clustering similarity between (**a**) and (**b**).

(**c**-**n**) Visualization of the dispersion of olfactory sensory neuron populations on the UMAP plot reported in (**a**, left) and (**b**, right).

Source data are provided as a Source Data file.

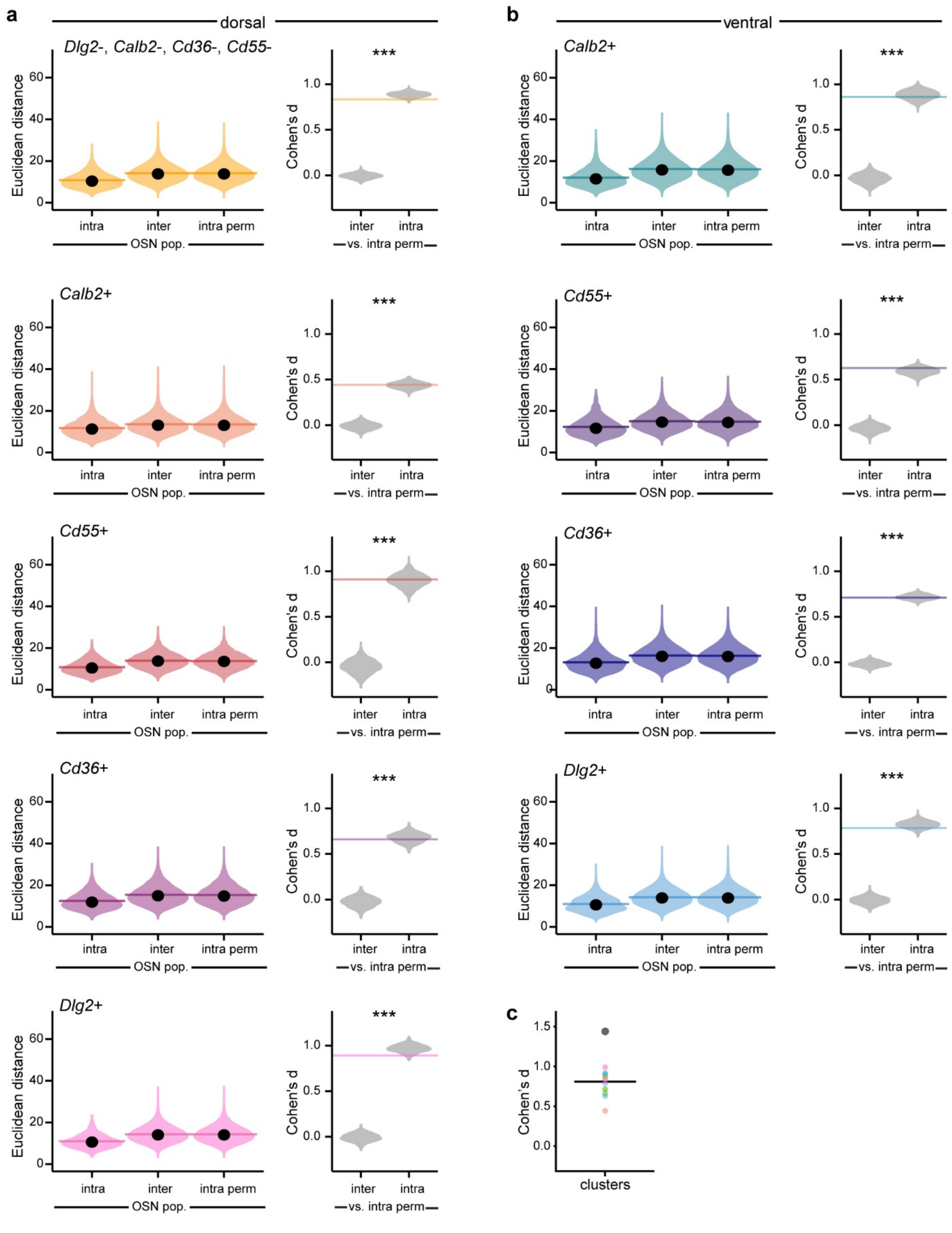

Supplementary Figure 5. Transcriptomic Euclidean distances between OSNs computed per cluster

(**a**-**b**) (left) Violin plots showing, per cluster, the density distribution of transcriptomic Euclidean distances (computed on the first 15 PCs) between pairs of OSNs expressing the same receptor (intra), different receptors (inter), and the same receptor after permutation of all receptor identities prior to distance calculation (intra perm, see methods). Horizontal bars correspond to mean values and dots correspond to median values. (right) Violin plots showing, per cluster, the range of Cohen's d values when comparing intra or inter OSN population pairwise transcriptomic distances with each of the

distributions of distances after permutation of receptor identities (n = 1000 per violin plot). The horizontal bar corresponds to the Cohen's d value computed between the distributions of intra and inter OSN population pairwise transcriptomic distances. *p<0.05; **p<0.01; ***p<0.001; two-sided two-sample Kolmogorov-Simonov test with Bonferroni correction. Exact p-values are provided in Supplementary Table 1.
(**c**) Dot plot showing the Cohen's d values per OSN cluster (colored dots) and for all OSN clusters (grey dot). The black horizontal bar corresponds to the median value.
Source data are provided as a Source Data file.

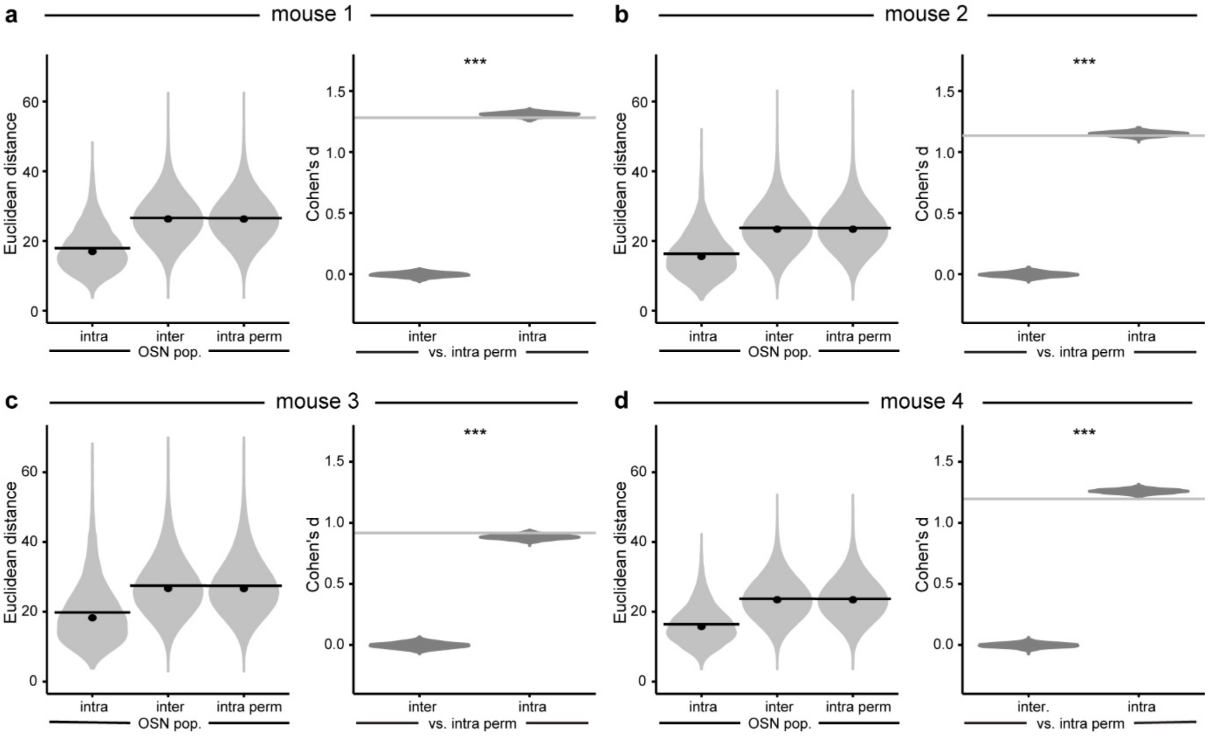

Supplementary Figure 6. Transcriptomic Euclidean distances between OSNs computed per mouse

(**a**-**d**) (left) Violin plots showing, per mouse, the density distribution of transcriptomic Euclidean distances (computed on the first 14 PCs for mouse 1, 12 PCs for mouse 2, 10 PCs for mouse 3 and 14 PCs for mouse 4) between pairs of OSNs expressing the same receptor (intra), different receptors (inter), and the same receptor after permutation of all receptor identities prior to distance calculation (intra perm, see methods). Horizontal bars correspond to mean values and dots correspond to median values. (right) Violin plots showing, per mouse, the range of Cohen's d values calculated between the distribution of intra or inter OSN population pairwise transcriptomic Euclidean distances and each of the distributions of distances after permutation of receptor identities (n = 1000 per violin plot). The horizontal bar corresponds to the Cohen's d value computed between the distributions of intra and inter OSN population pairwise transcriptomic Euclidean distances. *p<0.05; **p<0.01; ***p<0.001; two-sided two-sample Kolmogorov-Simonov test with Bonferroni correction. Exact p-values are provided in Supplementary Table 1.

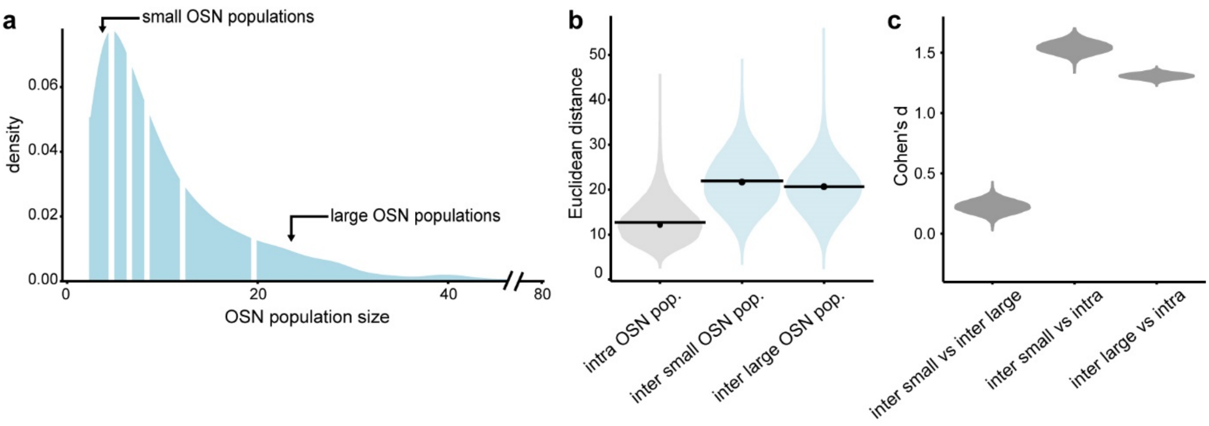

Supplementary Figure 7. Evaluation of the impact of dataset integration on transcriptomic Euclidean distances between OSNs

(**a**) Density distribution of OSN population sizes in the scRNA-seq dataset. Vertical white lines separate the density distribution into 6 equal frequency bins.

(**b**) Violin plots showing the density distribution of transcriptomic Euclidean distances (computed on the first 15 PCs) between all pairs of OSNs expressing the same receptor (intra, n = 97,999), pairs of OSNs pertaining to small OSN populations expressing different receptors (inter small, n = 166,036), and pairs of OSNs pertaining to large OSN populations expressing different receptors (inter large, n = 7,622,765).

(**c**) Violin plots showing the range of Cohen's d values calculated between the distinct distributions in (**b**) after subsampling 1/3 of the OSNs per population from the small and large populations (n = 10,000 per violin plot). Only inter OSN population transcriptomic Euclidean distances were subsampled.

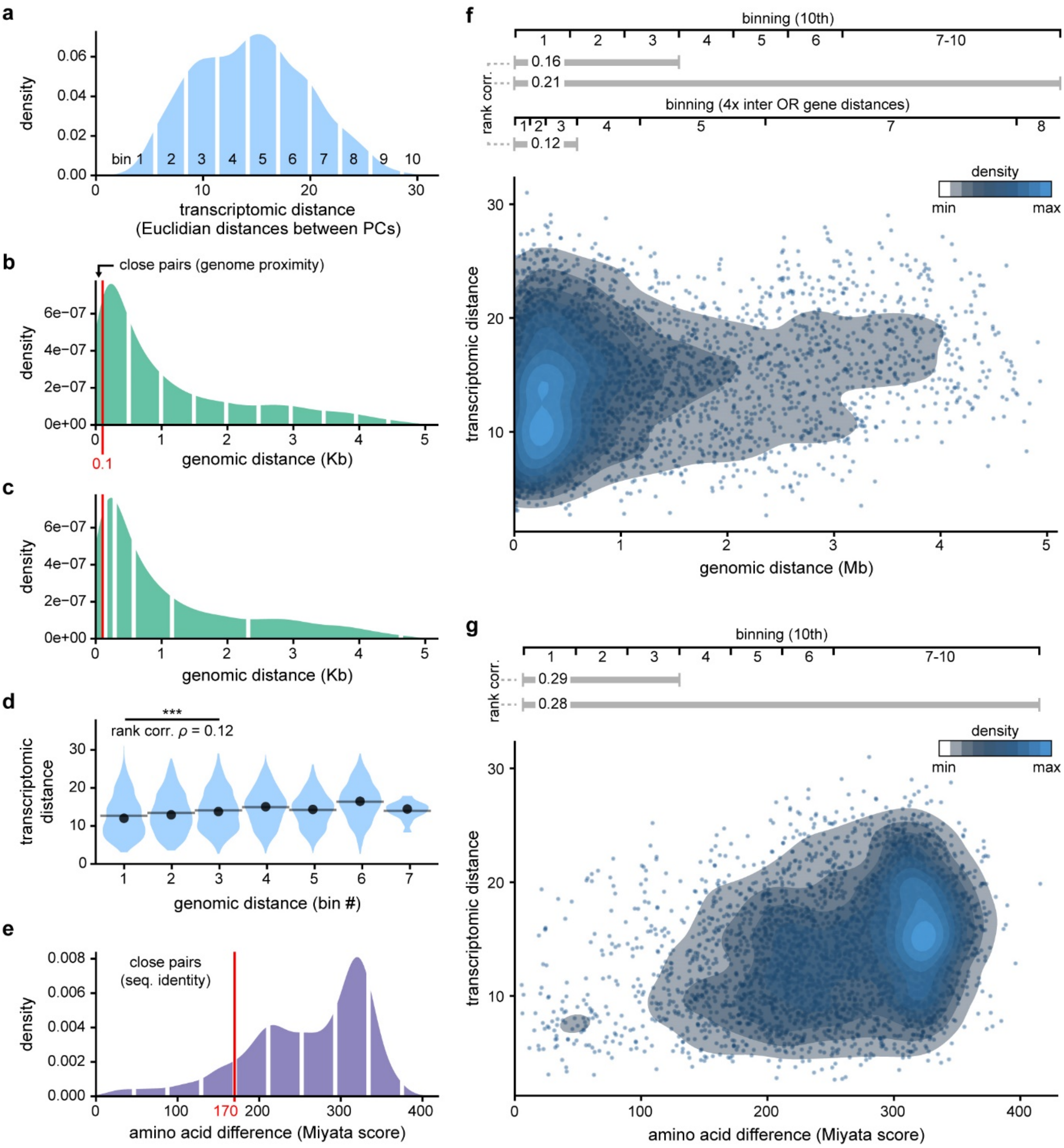

Supplementary Figure 8. Density distributions of pairwise distance metrics

(**a**) Density distribution of pairwise transcriptomic distances for all pairs of OSN populations expressing ORs from the same class and the same OR gene cluster (n=3602 pairs). White lines delineate bins used in (Fig. 2e,f), cutting the value range in 10 windows of equivalent width.

(**b**) Same as (**a**) but for genomic distance. The red line indicates the 95th percentile of this distribution, which is the maximum genomic distance of a pair to be considered close in terms of genomic proximity.

(**c**) Same distribution as in (**b**) but with a binning based on four times the average intergenic distance between adjacent OR genes. The subsequent limits are calculated as a multiplication by two of the precedent value.

(**d**) Pairwise transcriptomic distance distribution for each range of pairwise genomic distance values defined by the binning described in (**c**). Spearman's rank correlation score (ρ) was calculated for values comprised in the 3 first bins: Spearman's rank correlation ρ =0.12, ***p<0.001, n=1710 pairs.

(**e**) Density distribution of pairwise amino acid differences for all pairs of ORs from the same class and the same OR gene cluster. White lines delineate bins, which cut the value range in 10 windows of equivalent width. The red line indicates the fifth percentile of the data, which is the threshold to separated close and distant pairs in terms of amino acid difference.

(**f**) Correlation of genomic distance and transcriptomic distance for all pairs of OSN populations as described in (**a**). Binning windows are shown above for the two different methods (**b** and **c**). Spearman's rank correlation score *ρ* are given either for the three first bins or for the entire dataset (grey lines). Exact p-values can be found in Supplementary Table 1. Source data are provided as a Source Data file.

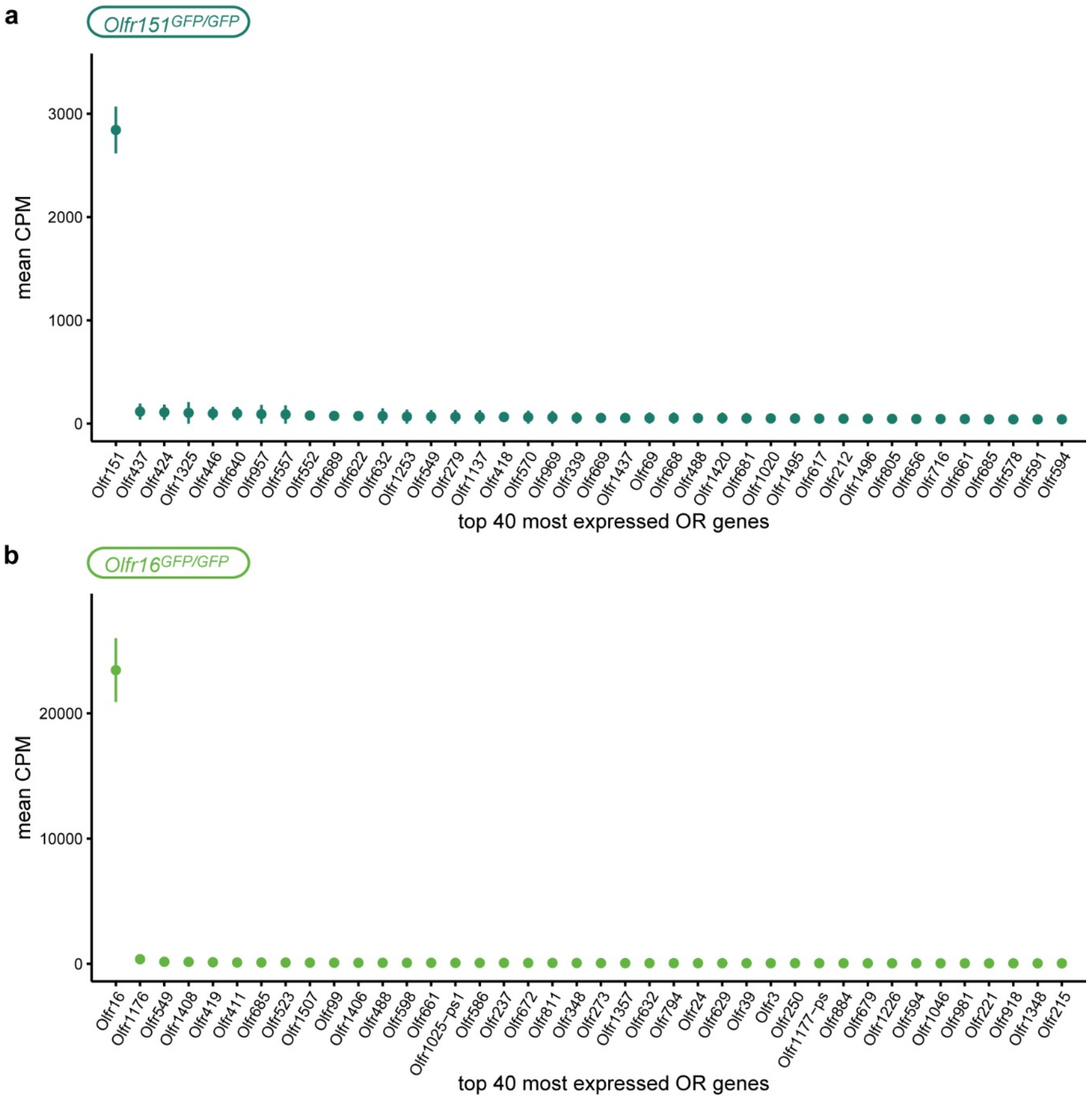

Supplementary Figure 9. Normalized counts mapped to OR genes in FAC-sorted *Olfr151*- and *Olfr16*-expressing populations.

(**a**) Mean normalized read counts per million (mean CPM) of the 40 most expressed OR genes detected in the FACS-seq of the *Olfr151*-transcribing population. Each dot represents the mean of n=3 samples (each sample is constituted by a pool of OSNs from n=4 animals). Horizontal bars centered on each data represent +/- SEM.

(**b**) Same as (**a**) but for the FACS-seq of the *Olfr16*-transcribing population.

Source data are provided as a Source Data file.

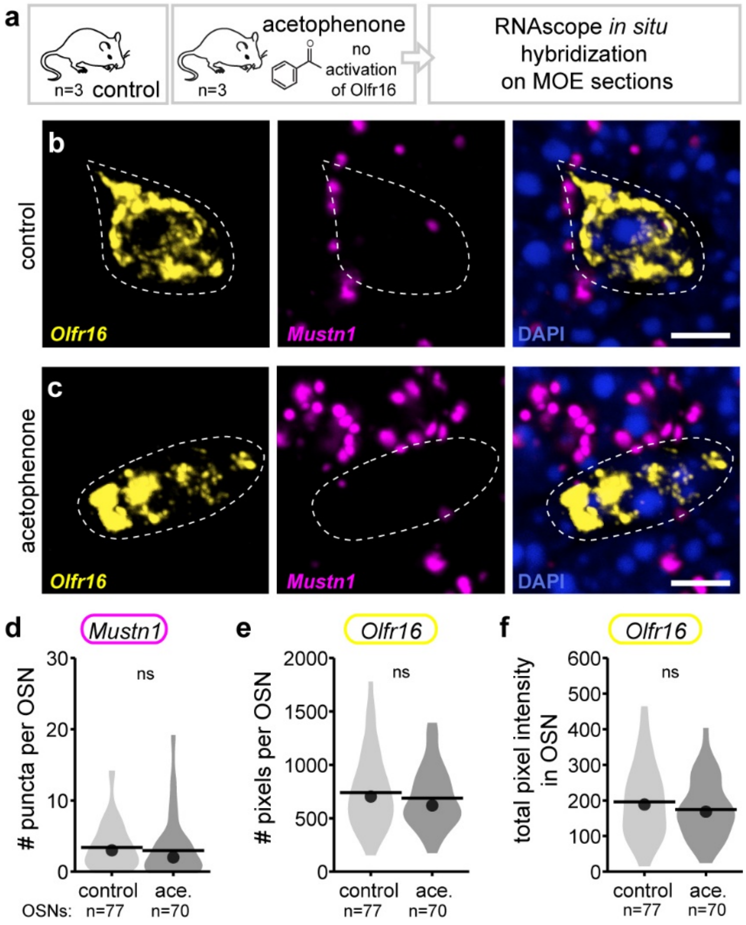

Supplementary Figure 10. Odorant-induced transcriptomic modulations are specific to cells expressing the activated OR.

(**a**) Schematic of the experiment. After being exposed to acetophenone for 5 hours, coronal sections of the main olfactory epithelium (MOE) were processed for RNAscope *in situ* hybridization. This ligand is known for not activating Olfr16.

(**b**-**c**) Magnification of a cell expressing *Olfr16* in the MOE of a control mouse (**b**) and in a mouse exposed to acetophenone (**c**). Cells were labeled with probes against *Olfr16* (yellow) and *Mustn1* (magenta) and counterstained with DAPI (blue). Scale bar, 5 µm.

(**d**-**f**) Quantification of *Mustn1* (**d**) and *Olfr16* (**e** and **f**) transcription. Horizontal bars correspond to mean values and dots correspond to median values. Two-sided Wilcoxon signed rank test. Exact p-values are provided in Supplementary Table 1.

Source data are provided as a Source Data file.

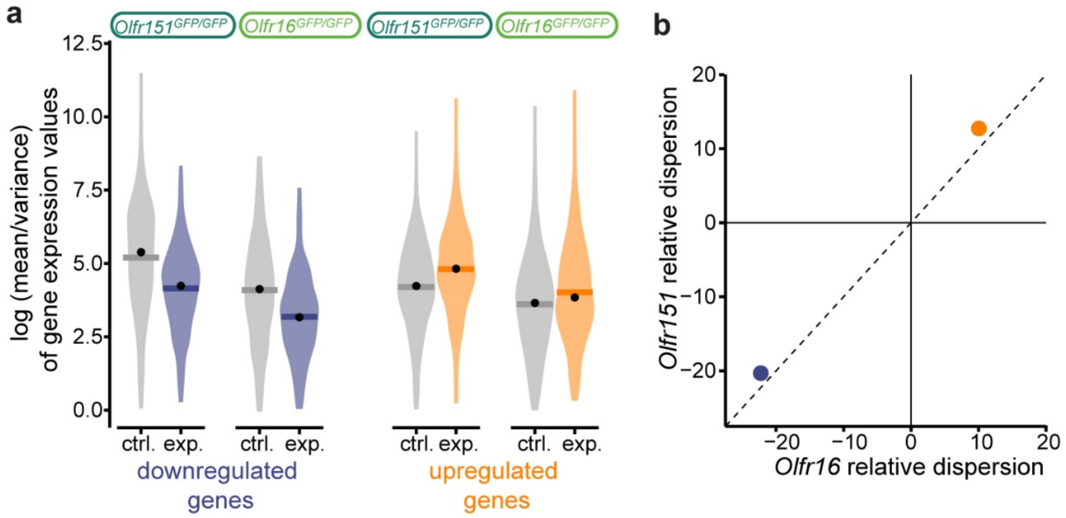

Supplementary Figure 11. Dispersion analysis of the modulated genes in *Olfr151*$^{GFP/GFP}$ and *Olfr16*$^{GFP/GFP}$ populations

(**a**) Dispersion (i.e. log(variance/mean)) of gene expression levels in *Olfr151*$^{GFP/GFP}$ and *Olfr16*$^{GFP/GFP}$ populations, with or without odorant exposure. The left violin plots (purple) correspond to genes significantly downregulated after odorant exposure. The right violin plots (orange) correspond to genes significantly upregulated after odorant exposure. Grey violin plots show the distribution of the same genes in the corresponding control samples.

(**b**) Dispersion modulation in the exposed samples relative to the non-exposed samples, calculated as the mean of the dispersion in the exposed samples divided by the mean of the dispersion of the non-exposed samples in the modulated genes. Blue dot: relative dispersion of the downregulated genes. Orange dot: relative dispersion of the upregulated genes.

Source data are provided as a Source Data file.

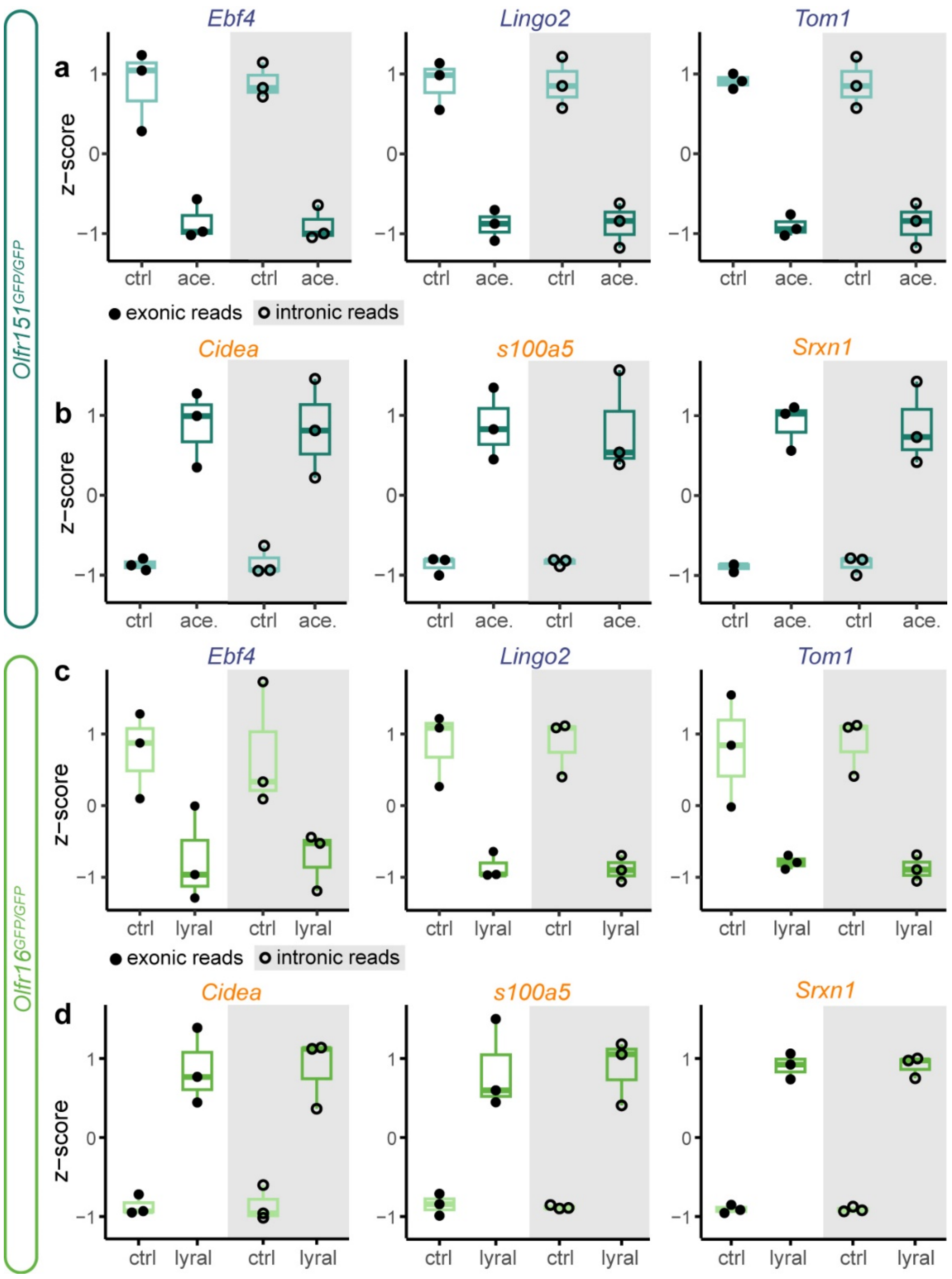

Supplementary Figure 12. Odorant-induced modulations of mRNA levels result from transcriptional regulation

(**a**-**b**) Scaled normalized expression levels (z-score) of mature and nascent RNA of a selected set of downregulated (**a**) and upregulated (**b**) genes in *Olfr151*-expressing neurons after acetophenone exposure. Mature mRNA levels are quantified with exonic reads (filled dots, white background), nascent RNA levels are quantified with intronic reads (empty dots, shaded background) in the control (ctrl) and exposed (ace.) samples. Each dot corresponds to one sample (which is constituted by a pool of OSNs from n=4 mice). Boxplot whiskers correspond to the minimum and maximum values, box limits represent Q1 to Q3, the line represents the mean.

(**c**-**d**) Same as (**a**) and (**b**) but in *Olfr16*-expressing neurons after lyral exposure.

Source data are provided as a Source Data file.

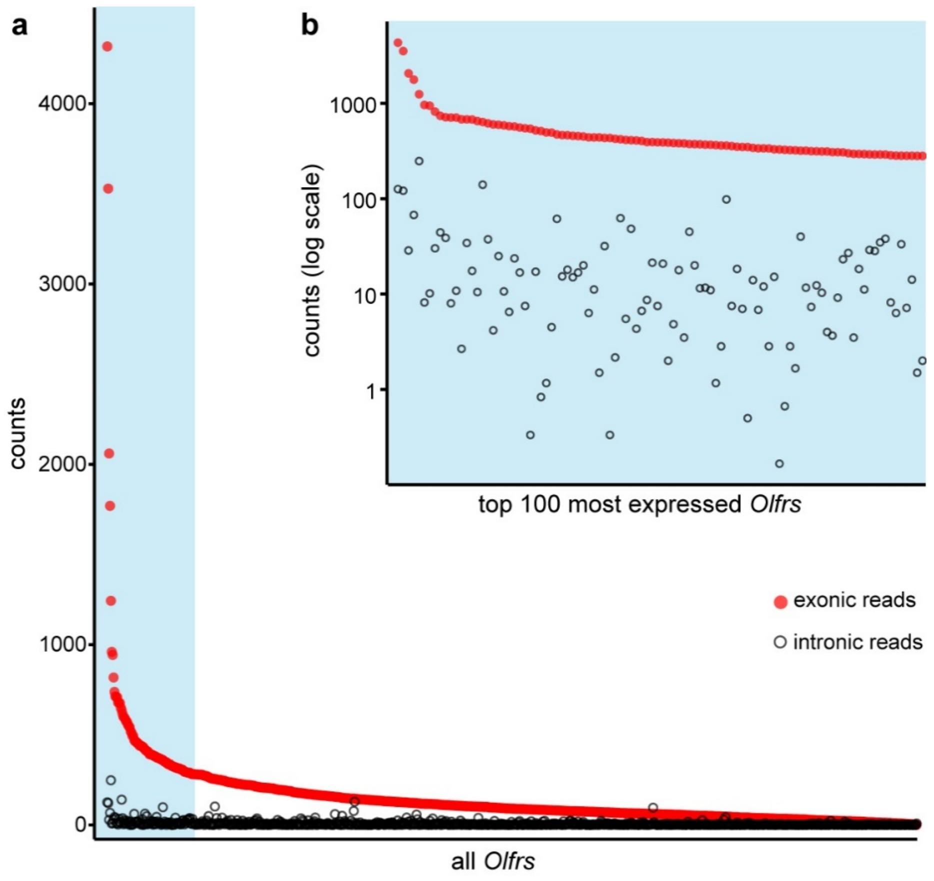

Supplementary Figure 13. Detection power of mature and nascent transcripts in our bulk RNA-seq experiment
(**a**) Exonic OR gene mean read counts (red dots) and intronic OR gene mean read counts (black circles) for all 904 OR transcripts detected in our bulk RNA-seq, ordered by mean exonic counts. The blue area is shown in (**b**).
(**b**) Top 100 OR genes shown in (**a**), featuring the same data but displayed with a logarithmic scaling of the y-axis.
Source data are provided as a Source Data file.

Supplementary Table 1

Statistics						
**Figure**	**Description**	**Sample size**	**Statistical test**	**Treatment effect**	**P value**	**Significance**
1e top	Cohen's d distribution comparison	1000 per distribution	Two-sample Kolmogorov-Smirnov test	D=1	<2.2e-16	***
1e bottom	Cohen's d distribution comparison	1000 per distribution	Two-sample Kolmogorov-Smirnov test	D=1	<2.2e-16	***
Suppl. 5a-b	Cohen's d distribution comparison with Bonferroni correction	1000 per distribution	Two-sample Kolmogorov-Smirnov test	D=1	<2.2e-15 for all (10 tests)	***
Suppl. 6a-c	Cohen's d distribution comparison with Bonferroni correction	1000 per distribution	Two-sample Kolmogorov-Smirnov test	D=1	<2.2e-15 for all (4 tests)	***
2e	Correlation between genomic distance and transcriptomic distance (3 first bins of genomic distances)	3602 pairs of OSN populations	Spearman's rank correlation	Rho = 0.1563775	1.091e-15	***
2e	Correlation amino acid difference and transcriptomic distance (3 first bins of amino acid distances)	222 pairs of OSN populations	Spearman's rank correlation	Rho = 0.2917749	1.084e-05	***
Suppl. 8d	Correlation between genomic distance and transcriptomic distance (3 first bins of genomic distances, alternative binning)	1710 pairs of OSN populations	Spearman's rank correlation	Rho = 0.12103	5.177e-07	***
2g	Transcriptomic distance, close(genome)-close(aa) vs distant(genome)-close(aa)	n=200, 27 pairs of OSN populations	Wilcoxon rank sum test with continuity correction	W = 3330	0.148 (adjusted)	ns
2g	Transcriptomic distance, distant(genome)-close(aa) vs distant(genome)-distant(aa)	n=27, 57796 pairs of OSN populations	Wilcoxon rank sum test with continuity correction	W = 183217	5.78e-12 (adjusted)	***
2g	Transcriptomic distance, close(genome)-close(aa) vs close(genome)-distant(aa)	n=200, 236 pairs of OSN populations	Wilcoxon rank sum test with continuity correction	W = 17254	1.3e-6 (adjusted)	***
3r	Mustn1 #puncta per OSN, control vs lyral exposure	n=62, 53 cells	Wilcoxon rank sum test with continuity correction	W = 799.5	1.947e-06	***

3s	Olfr16 #pixels per OSN, control vs lyral exposure	n=62, 53 cells	Wilcoxon rank sum test with continuity correction	W = 2399	2.245e-05	***
3t	Olfr16 total pixel intensity in OSN, control vs lyral exposure	n=62, 53 cells	Wilcoxon rank sum test with continuity correction	W = 2289.5	2.894e-4	***
Suppl. 10d	Mustn1 #puncta per OSN, control vs aceto. exposure	n=77, 70 cells	Wilcoxon rank sum test with continuity correction	W = 2265.5	0.09289	ns
Suppl. 10e	Olfr16 #pixels per OSN, control vs aceto. exposure	n=77, 70 cells	Wilcoxon rank sum test with continuity correction	W = 2383	0.227	ns
Suppl. 10f	Olfr16 total pixel intensity in OSN, control vs aceto. exposure	n=77, 70 cells	Wilcoxon rank sum test with continuity correction	W = 2503	0.4576	ns
4j	GO terms	NA	Fisher's exact test, Benjamini-Hochberg correction	NA	NA	NA
5c	Ebf4 - intronic<br>Ebf4 - exonic	n=3,3 samples	Two-sample independent t-test, FDR adjusted p-values	t = -9.913 df=4<br>t=-5.273 df=4	8.72e-04<br>6.20e-03	***<br>**
5c	Lingo2 – intronic<br>Lingo2 - exonic	n=3,3 samples	Two-sample independent t-test, FDR adjusted p-values	t = -7.14 df=4<br>t = -8.556 df=4	2.04e-03<br>1.54e-03	**<br>**
5c	Tom1 – intronic<br>Tom1 - exonic	n=3,3 samples	Two-sample independent t-test, FDR adjusted p-values	t = -13.64 df=4<br>t = -19.02 df=4	5.01e-04<br>1.35e-04	***<br>***
5d	Cidea – intronic<br>Cidea - exonic	n=3,3 samples	Two-sample independent t-test, FDR adjusted p-values	t = 4.481 df=4<br>t = 6.293 df=4	1.10e-02<br>3.26e-03	*<br>**
5d	S100a5 - intronic<br>S100a5 - exonic	n=3,3 samples	Two-sample independent t-test, FDR adjusted p-values	t = 4.494 df=4<br>t = 6.507 df=4	1.10e-02<br>3.26e-03	*<br>**
5d	Srxn1 - intronic<br>Srxn1 - exonic	n=3,3 samples	Two-sample independent t-test, FDR adjusted p-values	t =5.628 df=4<br>t =10.457 df=4	1.10e-02<br>1.42e-03	*<br>**
5f	Ebf4 - intronic<br>Ebf4 - exonic	n=3,3 samples	Two-sample independent t-test, FDR adjusted p-values	t =-2.555 df=4<br>t =-2.897 df=4	6.30e-02<br>4.42e-02	*<br>*
5f	Lingo2 – intronic<br>Lingo2 - exonic	n=3,3 samples	Two-sample independent t-test, FDR adjusted p-values	t =-7.071 df=4<br>t =-5.421 df=4	3.58e-03<br>1.68e-02	**<br>*
5f	Tom1 – intronic<br>Tom1 - exonic	n=3,3 samples	Two-sample independent t-test, FDR adjusted p-values	t =-6.841 df=4<br>t =-3.473 df=4	3.58e-03<br>3.83e-02	**<br>*
5g	Cidea – intronic<br>Cidea - exonic	n=3,3 samples	Two-sample independent t-test, FDR adjusted p-values	t =6.054 df=4<br>t =6.046 df=4	3.76e-03<br>5.66e-03	**<br>**
5g	S100a5 - intronic<br>S100a5 - exonic	n=3,3 samples	Two-sample independent t-test, FDR adjusted p-values	t =7.332 df=4<br>t =5.015 df=4	2.76e-03<br>7.41e-03	**<br>**
5g	Srxn1 - intronic	n=3,3 samples	Two-sample independent t-test,	t =22.412 df=4	7.04e-05	***

	Srxn1 - exonic		FDR adjusted p-values	t =18.419 df=4	1.53e-04	***
5i	Olfr741 - intronic<br><br>Olfr741 - exonic	n=3,3 samples	Two-sample independent t-test, FDR adjusted p-values	t =0.346 df=4<br>t =0.176 df=4	9.18e-01<br><br>8.87e-01	ns<br><br>ns
5i	Olfr1364 - intronic<br><br>Olfr1364 - exonic	n=3,3 samples	Two-sample independent t-test, FDR adjusted p-values	t =0.289 df=4<br>t =-0.151 df=4	9.18e-01<br><br>8.87e-01	ns<br><br>ns
5i	Olfr15 - intronic<br><br>Olfr15 - exonic	n=3,3 samples	Two-sample independent t-test, FDR adjusted p-values	t =-0.044 df=4<br>t =-0.36 df=4	9.67e-01<br><br>8.87e-01	ns<br><br>ns
5i	Olfr60 - intronic<br><br>Olfr60 - exonic	n=3,3 samples	Two-sample independent t-test, FDR adjusted p-values	t =-7.355 df=4<br>t =-14.48 df=4	6.37e-03<br><br>6.05e-04	**<br><br>***
5i	Olfr169 - intronic<br><br>Olfr169 - exonic	n=3,3 samples	Two-sample independent t-test, FDR adjusted p-values	t = -7.466 df=4<br>t =-8.971 df=4	6.37e-03<br><br>1.99e-03	**<br><br>**
5i	Olfr166 - intronic<br><br>Olfr166 - exonic	n=3,3 samples	Two-sample independent t-test, FDR adjusted p-values	t = -3.246 df=4<br>t =-13.53 df=4	7.35e-02<br><br>6.05e-04	*<br><br>***
5i	Olfr171 - intronic<br><br>Olfr171 - exonic	n=3,3 samples	Two-sample independent t-test, FDR adjusted p-values	t =-2.107 df=4<br>t =-3.515 df=4	1.80e-01<br><br>4.30e-02	ns<br><br>*
5j	Fluorescence exposed/mean control	n=128 OSNs	Unpaired two-tailed t-test with Welch's correction	t =3.176, df=221.2	0.0017	**

Supplementary Table 2

GO terms			
**GO term ID**	**Description**	**GO term type**	**Adjusted p-value**
GO:0001667	Ameboidal-type cell migration	BP	4.38e-02
GO:0003008	System process	BP	2.39e-02
GO:0003013	Circulatory system process	BP	2.03e-02
GO:0007154	Cell communication	BP	2.03e-02
GO:0023052	Signaling	BP	9.30e-03
GO:0051239	Regulation of multicellular organismal process	BP	2.39e-02
GO:1902547	Regulation of cellular response to vascular endothelial growth factor stimulus	BP	4.48e-02
GO:0004930	G protein-coupled receptor activity	MF	2.39e-02
GO:0045499	Chemorepellent activity	MF	2.39e-02
GO:0005576	Extracellular region	CC	2.36e-03
GO:0005886	Plasma membrane	CC	4.57e-04
GO:0005887	Integral component of plasma membrane	CC	2.39e-02
GO:0016020	Membrane	CC	9.30e-03
GO:0016021	Integral component of membrane	CC	2.03e-02
GO:0031224	Intrinsic component of membrane	CC	1.40e-02
GO:0031226	Intrinsic component of plasma membrane	CC	2.39e-02
GO:0071944	Cell periphery	CC	1.30e-04
