# Supplementary material for: Transcriptional adaptation of olfactory sensory neurons to GPCR identity and activity
Source: Nat Commun. 2022 May 25;13:2929. doi: 10.1038/s41467-022-30511-4 (PMC9132991; doi:10.1038/s41467-022-30511-4)
Supplement: Supplementary file 2 — Reporting Summary [file 41467_2022_30511_MOESM2_ESM.pdf]

nature portfolio

Corresponding author(s): Ivan Rodriguez
Alan Carleton

Last updated by author(s): 2022 04 29

Reporting Summary

Nature Portfolio wishes to improve the reproducibility of the work that we publish. This form provides structure for consistency and transparency in reporting. For further information on Nature Portfolio policies, see our Editorial Policies and the Editorial Policy Checklist.

Statistics

For all statistical analyses, confirm that the following items are present in the figure legend, table legend, main text, or Methods section.

n/a	Confirmed	
[ ]	[x]	The exact sample size (n) for each experimental group/condition, given as a discrete number and unit of measurement
[ ]	[x]	A statement on whether measurements were taken from distinct samples or whether the same sample was measured repeatedly
[ ]	[x]	The statistical test(s) used AND whether they are one- or two-sided<br>*Only common tests should be described solely by name; describe more complex techniques in the Methods section.*
[ ]	[x]	A description of all covariates tested
[ ]	[x]	A description of any assumptions or corrections, such as tests of normality and adjustment for multiple comparisons
[ ]	[x]	A full description of the statistical parameters including central tendency (e.g. means) or other basic estimates (e.g. regression coefficient) AND variation (e.g. standard deviation) or associated estimates of uncertainty (e.g. confidence intervals)
[ ]	[x]	For null hypothesis testing, the test statistic (e.g. F, t, r) with confidence intervals, effect sizes, degrees of freedom and P value noted<br>*Give P values as exact values whenever suitable.*
[x]	[ ]	For Bayesian analysis, information on the choice of priors and Markov chain Monte Carlo settings
[x]	[ ]	For hierarchical and complex designs, identification of the appropriate level for tests and full reporting of outcomes
[ ]	[x]	Estimates of effect sizes (e.g. Cohen's d, Pearson's r), indicating how they were calculated

Our web collection on statistics for biologists contains articles on many of the points above.

Software and code

Policy information about availability of computer code

Data collection	Single cell RNA-seq data was collected using 10X Genomic Chromium technology. Bulk RNA-seq data was sequenced by Illumina Hiseq 2500 and Illumina Hiseq 4000 for Olfr16 and Olfr151 experiments respectively.
Data analysis	For the single-cell RNA-seq data, reads were mapped and counted using Cell Ranger version 6.0.1 and Seurat R package version 4.0.1. Cohen's d values were computed using the effetsize R package version 0.8.1. The kneed python package version 0.7.0 was used to select the number of PCs used for UMAP plot generation, clustering and Euclidean distance calculation for the mature OSN dataset. For bulk RNA-seq, reads were mapped to the reference genome (GRCm38) using STAR version 2.7.0. Gene expression quantification was carried out using featureCounts version 1.6.3. DESeq2 package version 1.30.1 was used to perform differential expression analysis with the Wald test used for significance. Then, the p values were adjusted for multiple comparisons using the Benjamini-Hochberg procedure. Fold-changes of differentially expressed genes were estimated using the apeglm R package version 1.16.0.<br>All original code used to analyze data reported in the paper are provided at the GitHub repository https://github.com/irlabgenev/DREAM_pt2.<br>In situ hybridization images were analyzed with Image J version 1.53q. RNAscope in situ hybridization images were analyzed with CellProfiler software version 4.2.1.

For manuscripts utilizing custom algorithms or software that are central to the research but not yet described in published literature, software must be made available to editors and reviewers. We strongly encourage code deposition in a community repository (e.g. GitHub). See the Nature Portfolio guidelines for submitting code & software for further information.

Data

Policy information about availability of data

All manuscripts must include a data availability statement. This statement should provide the following information, where applicable:

- Accession codes, unique identifiers, or web links for publicly available datasets
- A description of any restrictions on data availability
- For clinical datasets or third party data, please ensure that the statement adheres to our policy

Bulk and single-cell RNA-seq data have been deposited in NCBI GEO and can be accessed using the superseries GSE185168 accession number, and are publicly available as of the date of publication. Our custom annotation of the mouse genome GRCm38 as well as the OR protein phylogeny have been deposited in figshare [https://doi.org/10.6084/m9.figshare.c.5957625].

Field-specific reporting

Please select the one below that is the best fit for your research. If you are not sure, read the appropriate sections before making your selection.

- [x] Life sciences
- [ ] Behavioural & social sciences
- [ ] Ecological, evolutionary & environmental sciences

For a reference copy of the document with all sections, see nature.com/documents/nr-reporting-summary-flat.pdf

Life sciences study design

All studies must disclose on these points even when the disclosure is negative.

Sample size

For the single-cell RNA-seq, we found that due to the consistency among the samples, 4 animals were sufficient to capture over 65% of all OSN populations represented by at least 3 cells in the dataset (from the 1,200 OSN populations in the mouse, 798 were analyzed in the current study) and generate robust results. For FACS-seq, for each of the 4 conditions we analyzed 3 samples which were each constituted by cells from 4 mice (1). For bulk RNA-seq, 3 animals per condition were used (1). For RNAscope in situ hybridization, 3 male mice per condition were analyzed. For In situ hybridization, 4 male mice per condition were analyzed (2). Sample size determination was always based on established protocols and according to the veterinary guidelines and regulations of the University and of the state of Geneva.

(1) von der Weid et al. Large-scale transcriptional profiling of chemosensory neurons identifies receptor-ligand pairs in vivo. Nat Neurosci. 2015 Oct;18(10):1455-63. doi: 10.1038/nn.4100

(2) Erben L, Buonanno A. Detection and Quantification of Multiple RNA Sequences Using Emerging Ultrasensitive Fluorescent In Situ Hybridization Techniques. Curr Protoc Neurosci. 2019;87(1):e63. doi:10.1002/cpns.63

Data exclusions

For the single-cell RNA-seq data, cells were excluded from the analysis based on parameters described in the methods. For the main olfactory epithelium dataset, in addition to the removal of immune, blood and suffering cells (i.e. cells with a high percentage of mitochondrial gene counts), cells were filtered out if their percentage of mitochondrial counts exceeded 10% of their total counts or if they expressed less than 1,000 genes. These values were tailored for the current dataset (data not shown). For the mature OSN dataset, mature OSNs were selected from the main olfactory epithelium dataset for downstream analyses and were subjected to further cell filtering. For each mature OSN, the detected olfactory receptors were ordered based on their expression levels. To remove cells that could correspond to multiplets (among those co-expressing multiple olfactory receptors), the distribution of the expression levels of the highest expressed receptors was analyzed using the log normalized data. An “is expressed” cutoff was set at three median absolute deviations from the median of the levels of expression of the highest expressed receptors. OSNs whose highest expressed receptor had an expression level below this cutoff and OSNs that expressed more than one receptor at an expression level higher than this cutoff were removed from the dataset. Moreover, mature OSNs that did not show receptor expression were discarded from the dataset. Finally, OSN populations represented by less than 3 cells in the dataset were also discarded.

Replication

Number of replicates for each experiment are indicated in the figure, figure legend and methods.

Randomization

For all experiments where there were different conditions, all mice were randomly attributed to a condition.

Blinding

For the measurement of puncta and fluorescence intensity in olfactory sensory neurons, the experimenter was blind to the condition. For all other analyses blinding was not relevant as the analyses were automatic and the same parameters were applied for all conditions.

Reporting for specific materials, systems and methods

We require information from authors about some types of materials, experimental systems and methods used in many studies. Here, indicate whether each material, system or method listed is relevant to your study. If you are not sure if a list item applies to your research, read the appropriate section before selecting a response.

Materials & experimental systems

n/a	Involved in the study	
[ ]	[x]	Antibodies
[x]	[ ]	Eukaryotic cell lines
[x]	[ ]	Palaeontology and archaeology
[ ]	[x]	Animals and other organisms
[x]	[ ]	Human research participants
[x]	[ ]	Clinical data
[x]	[ ]	Dual use research of concern

Methods

n/a	Involved in the study	
[x]	[ ]	ChIP-seq
[x]	[ ]	Flow cytometry
[x]	[ ]	MRI-based neuroimaging

Antibodies

Antibodies used	Anti-Fluorescein-POD, Fab fragments (Roche, ref. 11426346910).
Validation	This antibody was designed to bind to fluorescein-labeled UTP in RNA probes (Roche, ref. 11175025910).

Animals and other organisms

Policy information about studies involving animals; ARRIVE guidelines recommended for reporting animal research

Laboratory animals	For scRNA-seq, 8 weeks old male C57BL/6J mice were used. For FACS-seq, 7 weeks old male Olfr16(GFP/GFP) and Olfr151(GFP/GFP) mice were used. For bulk RNA-seq, 8 weeks old male C57BL/6J mice were used. For in situ hybridization, 8 weeks old male C57BL/6J mice were used. For RNA scope in situ hybridization 5 weeks old male and female Olfr16(GFP/GFP) mice were used (equally distributed in control and exposed groups).
Wild animals	No wild animals were used.
Field-collected samples	No field collected samples were used.
Ethics oversight	All animals were housed and treated in accordance with the veterinary guidelines and regulations of the University and of the state of Geneva.

Note that full information on the approval of the study protocol must also be provided in the manuscript.
