# Supplementary material for: Transcriptional adaptation of olfactory sensory neurons to GPCR identity and activity
Source: Nat Commun. 2022 May 25;13:2929. doi: 10.1038/s41467-022-30511-4 (PMC9132991; doi:10.1038/s41467-022-30511-4)
Supplement: Supplementary file 3 — Description of Additional Supplementary Files [file 41467_2022_30511_MOESM3_ESM.pdf]

Title: Supplementary Data 1.
Description: Differentially expressed genes in Olfr16 and Olfr151- transcribing populations after agonist exposure. Fold change of the strongly modulated genes. Selection criteria: adjusted p-value < 0.1 and log2(fold change) < -0.5 (=downregulated genes) or adjusted p-value < 0.1 and log2(fold change) > 0.5 (=upregulated genes). P-values: Wald test (two-tailed), BenjaminiHochberg adjusted.

Title: Supplementary Data 2.
Description: Differentially expressed genes between Olfr151- and Olfr16-transcribing populations at basal state. Gene expression fold differences of genes identified as being differentially expressed between Olfr151- and Olfr16-transcribing populations, at basal state. Selection criteria: Adjusted p-value < 0.1 and fold difference > 4. P-values: Wald test (two-tailed), BenjaminiHochberg adjusted.

Title: Supplementary Data 3.
Description: Genes that were strongly modulated after activation in one OSN population and not in the other. Gene expression fold change after ligand exposure, and OSN population specificity assessment. Selection criteria: Adjusted p-value < 0.1, log2(fold change) < -2 (=downregulated genes); adjusted p-value < 0.1, log2(fold change) > 2 (=upregulated genes) P-values: Wald test (two-tailed), BenjaminiHochberg adjusted. Specific to the population: gene that was not detected in the other population.
